# *LncRNA-Smad7* mediates cross-talk between Nodal/TGF-β and BMP signaling to regulate cell fate determination of pluripotent and multipotent cells

**DOI:** 10.1093/nar/gkac780

**Published:** 2022-09-22

**Authors:** Xiaohui Kong, Kun Yan, Pujuan Deng, Haipeng Fu, Hongyao Sun, Wenze Huang, Shuangying Jiang, Junbiao Dai, Qiangfeng Cliff Zhang, Jun-jie Gogo Liu, Qiaoran Xi

**Affiliations:** MOE Key Laboratory of Protein Sciences, School of Life Sciences, Tsinghua University, Beijing 100084, China; Tsinghua-Peking Center for Life Sciences, School of Life Sciences, Tsinghua University, Beijing 100084, China; School of Life Sciences, Tsinghua-Peking Joint Center for Life Sciences, Beijing Advanced Innovation Center for Structural Biology, Tsinghua University, Beijing 100084, China; MOE Key Laboratory of Protein Sciences, School of Life Sciences, Tsinghua University, Beijing 100084, China; Joint Graduate Program of Peking-Tsinghua-NIBS, Tsinghua University, Beijing 100084, China; Tsinghua-Peking Center for Life Sciences, School of Life Sciences, Tsinghua University, Beijing 100084, China; MOE Key Laboratory of Bioinformatics, Beijing Advanced Innovation Center for Structural Biology & Frontier Research Center for Biological Structure, Center for Synthetic and Systems Biology, School of Life Sciences, Tsinghua University, Beijing 100084, China; CAS Key Laboratory of Quantitative Engineering Biology, Guangdong Provincial Key Laboratory of Synthetic Genomics and Shenzhen Key Laboratory of Synthetic Genomics, Shenzhen Institute of Synthetic Biology, Shenzhen Institute of Advanced Technology, Chinese Academy of Sciences, Shenzhen 518055, China; CAS Key Laboratory of Quantitative Engineering Biology, Guangdong Provincial Key Laboratory of Synthetic Genomics and Shenzhen Key Laboratory of Synthetic Genomics, Shenzhen Institute of Synthetic Biology, Shenzhen Institute of Advanced Technology, Chinese Academy of Sciences, Shenzhen 518055, China; Tsinghua-Peking Center for Life Sciences, School of Life Sciences, Tsinghua University, Beijing 100084, China; MOE Key Laboratory of Bioinformatics, Beijing Advanced Innovation Center for Structural Biology & Frontier Research Center for Biological Structure, Center for Synthetic and Systems Biology, School of Life Sciences, Tsinghua University, Beijing 100084, China; School of Life Sciences, Tsinghua-Peking Joint Center for Life Sciences, Beijing Advanced Innovation Center for Structural Biology, Tsinghua University, Beijing 100084, China; MOE Key Laboratory of Protein Sciences, School of Life Sciences, Tsinghua University, Beijing 100084, China

## Abstract

Transforming growth factor β (TGF-β) superfamily proteins are potent regulators of cellular development and differentiation. Nodal/Activin/TGF-β and BMP ligands are both present in the intra- and extracellular milieu during early development, and cross-talk between these two branches of developmental signaling is currently the subject of intense research focus. Here, we show that the Nodal induced *lncRNA-Smad7* regulates cell fate determination via repression of BMP signaling in mouse embryonic stem cells (mESCs). Depletion of *lncRNA-Smad7* dramatically impairs cardiomyocyte differentiation in mESCs. Moreover, *lncRNA-Smad7* represses *Bmp2* expression through binding with the *Bmp2* promoter region via (CA)_12_-repeats that forms an R-loop. Importantly, *Bmp2* knockdown rescues defects in cardiomyocyte differentiation induced by *lncRNA-Smad7* knockdown. Hence, *lncRNA-Smad7* antagonizes BMP signaling in mESCs, and similarly regulates cell fate determination between osteocyte and myocyte formation in C2C12 mouse myoblasts. Moreover, *lncRNA-Smad7* associates with hnRNPK in mESCs and hnRNPK binds at the *Bmp2* promoter, potentially contributing to *Bmp2* expression repression. The antagonistic effects between Nodal/TGF-β and BMP signaling via *lncRNA-Smad7* described in this work provides a framework for understanding cell fate determination in early development.

## INTRODUCTION

Transforming growth factor β (TGF-β) family proteins are key components of the intra- and extracellular stem cell milieu of embryonic and somatic stem cells. Nodal/Activin/TGF-β (hereafter, TGF-β) and BMP signaling each play diverse roles in controlling pluripotent and multipotent cell fates (1–3). These roles are determined through transcriptional regulation mediated by the R-SMADs–SMAD4 complex coordinated by lineage-specific master regulators and chromatin associated proteins/factors (4). Cross-talk between TGF-β and BMP signaling has been actively investigated in tissue homeostasis maintenance processes (3,5,6). For example, TGF-β signaling suppresses BMP signaling by up-regulating genes encoding BMP signaling antagonists (e.g.*CTGF*, *Gremlin*) (7–9), and vice versa (5,10). Although cross-talk in TGF-β and BMP signaling is inevitable in cell fate determination, the molecular mechanism responsible for this cross-talk remains elusive.

The heart is one of the earliest differentiating organs, and heart development requires stepwise regulation dependent on the precise control of genetic networks that involve a suite of cardiac-specific transcription factors (e.g. *HAND1*, *HAND2*, *NKX2.5*, *MESP1*) (11–15), chromatin modifiers (16–18), lncRNAs (19–21), and signaling pathways (22,23). For example, Nodal and BMP signaling regulates cardiomyocyte commitment (22,24,25). While fine-scale temporal control by BMP2 signaling specifies cardiomyocyte differentiation from mouse embryonic stem cells (mESCs) (23,26), Nodal regulates asymmetric morphogenesis of heart looping by modulating cell proliferation, differentiation, and extracellular matrix composition (27). Cardiomyocyte maturation is regulated by the controlled proliferation of cardiac progenitor cells, and changes in progenitor cell proliferation rates may cause trabeculation and left ventricular noncompaction (LVNC) (28–30). It is therefore plausible that cross-talk between the two signaling branches can also function in regulating cardiomyocyte differentiation.

Numerous lncRNAs are broadly expressed during early development (18,31–33), in which the TGF-β family plays a central role. These early development and tissue homeostasis lncRNAs are also known to be induced by the TGF-β family, such as *DIGIT* in mESCs (34,35), *TGFB2-AS1* in HaCaT cells (36), and *lncRNA-Smad7* in mouse breast cancer and C2C12 cells (37,38). Moreover, regulatory elements embedded in lncRNA transcripts have been shown to be crucial for cellular functions through association with other factors including proteins, DNAs and RNAs (39,40). However, whether and how lncRNAs participate in cross-talk between TGF-β and BMP signaling branches has not been documented.

In this work, we show that *lncRNA-Smad7*, which is divergently transcribed from the shared promoter of *Smad7* (37,89), is activated by Nodal signaling in mESCs. *LncRNA-Smad7* then regulates cardiomyocyte differentiation by repressing *Bmp2* expression, and its knockdown impairs the cardiomyocyte differentiation process. Importantly, *Bmp2* knockdown rescues these *lncRNA-Smad7* KD-related defects. In addition, *lncRNA-Smad7* represses *Bmp2* expression through binding to its promoter region via its (CA)_12_-repeats. Moreover, *lncRNA-Smad7* antagonizes BMP signaling in mESCs, resulting in cell fate determination between osteocyte and myocyte formation in mouse myoblast C2C12 cells. Finally, we identified hnRNPK as an interaction partner of *lncRNA-Smad7* in mESCs, and CUT&Tag assays showed that hnRNPK binds the *Bmp2* promoter, indicating that *lncRNA-Smad7* may repress *Bmp2* expression through recruitment of hnRNPK. These findings thus provide new insight into the role of lncRNAs in regulating cross-talk between Nodal/TGF-β and BMP signaling during early development.

## MATERIALS AND METHODS

### Animal experiments

Mice were obtained from Tsinghua University Laboratory Animals Resource Center. And mice were housed at 20–22°C (12 h:12 h light:dark cycles, 50–60% humidity). All mice we used had a C57BL/6 background including adult mice (female postnatal 14 days) and pregnant mice (female, day 17.5). Embryos were isolated at the developmental stages (E17.5). The dissected tissues were used for the qPCR to verified the gene expressions in different tissues. All animal experiments were carried out in accordance with institutional guidelines for animal welfare and approved by the Institutional Animal Care and Use Committee (IACUC) at Tsinghua University, China.

### Cell culture

Mouse ESCs E14Tg2a.IV were maintained in feeder-free condition on 0.1% gelatin-coated dishes at 37°C with 5% CO_2_. Basic ES cells were cultured in DMEM (Gibco, Cat. No. 8120287), 15% fetal bovine serum (Excell Bio, Cat. No. FND500), 1% penicillin/streptomycin (Biological Industries, Cat. No. 1948087), 1% non-essential amino acids (Hyclone), 1% l-glutamine (Biological Industries, Cat. No. 2008114), 1% sodium pyruvate (Sigma, Cat. No. RNBJ3675), 100 μM β-mercaptoethanol (Sigma), 103 U/ml LIF. HEK293T (human embryonic kidney cells) and C2C12 cells are cultured in DMEM supplemented with 10% FBS (Excell Bio, Cat. No. FCS500).

### Cell differentiation

Mouse EBs formation and ectodermal differentiation were carried out as described by the supplier (ATCC) and previously reported (6). 1.5 × 10^5^ cells/ml in EB media were seeded in low-attached 10-cm dish without LIF to initiate the differentiation. We passaged the cells at the ratio of 1:6 on day 2 and change the media every two days. Retinoic acid (RA) was added for ectodermal differentiation on day 2. Cells were harvested on day 0 and day 2 to day 6 for total RNA extraction during the differentiation.

Cardiomyocyte differentiation was initiated via EB formation induced by hanging ∼20 μl suspensions of drops, with roughly equivalent embryonic stem cell counts (2500 cells) (7). The drops were combined after 2 days and treated with human BMP4 (0.2 ng/ml, PEPROTECH, Cat No. 315–27), Activin A (8 ng/ml, R&D, Cat No. 338-AC), human VEGF (5 ng/ml, PEPROTECH, Cat No. 80710) and l-ascorbic Acid (1 mM, Sigma, Cat# A2218) for 32 h. EBs were then transferred to 0.1% gelatin-coated plates and incubated for 6–8 days. Cells were harvested for total RNA extraction at day 0, day 2, day 3.3, day 6, day 8 and day 10. The cells were collected for immunofluorescence staining at pH3 on day 3.3 (80 h) and cTnT on day 10.

Myogenesis differentiation of C2C12 cells was carried out in 2% fetal equine serum (Solarbio, Cat No. S9050) at the confluence of 80–90%, we changed the media every day and collected the cells on day 0, day 2, day 4 to day 6 of for total RNA extraction. Osteoblast differentiation of C2C12 cells was induced by 50 ng/ml hBMP4 at the confluence of 40–50%, we collected the cells every 6 h from hour 0 to hour 48 for total RNA extraction. Alternatively, osteoblast differentiation of C2C12 cells was initiated with 500 cells in 6-well plate by 50 ng/ml hBMP4 for up to 10 days, we change the media with hBMP4 every day and set up alkaline phosphatase staining (AP staining) on day 10.

### Rapid amplification of cDNA ends (RACE)

5’ and 3’ RACE was performed using the SMART RACE cDNA Amplification Kit (Clontech) according to the manufacturer's recommendations. The full length of *lncRNA-Smad7* was cloned by overlap PCR according to the sequence information of 5’ and 3’ of RACE. Here we used the 3’ CDS primer to initiate the 3’ RACE: AGCAGTGGTATCAACGCAGAGTAC(T)_30_ V N (N = A, C, G, or T; V = A, G or C). The sequence of other primers are listed in Supplementary Table S2.

### Measurement of *lncRNA-Smad7* Copy Numbers (in E14 and C2C12 cells)

The copy number of the diluted full length of *lncRNA-Smad7* was calculated by DNA/RNA Copy Number Calculator from website (http://endmemo.com/bio/dnacopynum.php). The linearized full length of *lncRNA-Smad7* was cloned from a plasmid DNA (PUC19) containing *lncRNA-Smad7* using primers listed in Supplementary Table S2. A serial dilution of the linearized full length of *lncRNA-Smad7* cDNA was used to qRT-PCR to generate a standard curve for *lncRNA-Smad7*. To measure the *lncRNA-Smad7* copy number in E14 or C2C12 cells, total RNA extracted from 1.5 × 10^6^ and 1.2 × 10^6^ cells of each line was reverse transcribed into cDNAs for qPCR analysis, and the copy number could be quantitated from the standard curve. The qPCR primers were listed in Supplementary Table S1.

### PiggyBac mediated over-expression

For *Smad2* or *Smad4* reintroduction in the *Smad2* KO or *Smad4* KO mESCs: sequences harboring the *Smad2* (Gene ID: 17126) or *Smad4* (Gene ID: 17128) UTR, and CDS regions were introduced into *Smad2* KO or *Smad4* KO mESCs (PMID: 3762693) by PB-CAG system. The sequences of *Smad2* and *Smad4* were synthesized by Junbiao Dai lab.

For *lncRNA-Smad7* overexpression: The full-length transcript of *lncRNA-Smad7* were retrieved and cloned from E14 cells according to the RACE data, inserted into the PiggyBac vector resistant to hygromycin, and driven by CAG promoter. The overexpression vectors were co-transfected with pBASE by lipofectamine 2000. After drug selection of hygromycin (Amresco, Cat. No. HK547), the over-expressed cells were collected for RNA extraction and qPCR analysis.

### Generation of *lncRNA-Smad7* knock-out and 4x polyA-knock-in (KIpA) cells

The short guide RNAs (sgRNAs) were inserted into BbsI-linearized pSpCas9 (BB)-2A-GFP (PX458) vector (Addgene# 48138) respectively. The 4× polyA transcription stop cassette was cloned into the pMD19-T (TaKaRa) donor vector. We co-transfected the PX458 and donor vector containing homologous arms and 4× polyA sequence at a ratio of 1:4. Cells were transfected and GFP-positive cells were FACS sorted as single cells into 96-well plate at 48 h after transfection. The cells were cultured for 2 weeks followed by PCR-based genotyping. One clone showing deletion of the targeted region in *lncRNA-Smad7* genomic DNA and two clones showing 4× polyA insertion were picked up for further analysis.

### Generation of shRNA mediated knock-down cells

Short hairpins (shRNAs) were designed by Public TRC Portal (the RNAi Consortium, Broad Institute) or as previously reported. Annealed oligonucleotides of shRNAs were cloned into AgeI and EcoRI restriction enzymes digested pLKO.1-puro lentiviral vector driven by U6 promoter. Scramble shRNA was used as the control shRNA. The shRNAs were transfected into HEK293T cells to package lentivirus by lipofectamine 2000 (Invitrogen, Cat# 2270695) followed by infections of mESCs or C2C12 cells. After drug selection, cells were collected for RNA extraction and qPCR analysis. Sequence of shRNAs for *lncRNA-Smad7*, *Bmp2* and *Hand1* were listed in Supplementary Table S2.

### Subcellular fractionation assay

Subcellular fractionation was performed as previously described with some subtle modifications (41). One 10-cm plate of mESCs were digested by trypsin, then spun down at 1200 rpm for 3 min to collect. We separated cytoplasmic, nucleoplasm and chromatin fractions and isolated them by TRIzol reagent (Ambion, 204409) to fully dissolve the fractions.

### Proliferation analysis

EBs of cardiomyocyte on day 3.3 (CM D3.3, a total of 80 h) were collected and fixed in 4% paraformaldehyde (PFA) at room temperature (RT). The EBs were collected to the 30% sucrose solution for 1–2 days to enable most EBs to sink to the bottom and embedded with O.C.T. compound. EB sections were permeabilized in 0.5% Triton X-100 in PBS (PBST) for 30 min at RT, incubated in primary antibody anti-pH3 overnight and AlexaFluor-488-conjugated secondary antibody for 1 h at RT. Signals were detected with A1/SIM/STORM-confocal (Nikon, 17018750). The antibodies used in this study are listed in Supplementary Table S4.

### Immunofluorescence

Immunofluorescence of cTnT: EBs were seeded on 35 mm confocal dish on day 3.3 and cultured to day 10. CMs were fixed with 4% PFA for 10 min, permeabilized with 0.5% Triton X-100 in PBS for 10 min, stained with the primary antibody cTnT overnight at 4°C and AlexaFluor-594-conjugated secondary antibody for 1 h at RT. Signals were detected with A1R confocal microscopy (A1/SIM/STORM, Nikon, 17018750; HD25, Nikon). The antibody used in this study are shown in Supplementary Table S4.

Immunofluorescence of MY-32 (Myosin Heavy Chain 1 Antibody): C2C12 cells were seeded on 35 mm confocal dish and induced to myogenesis differentiation with 2% fetal equine serum at the confluence of 80–90%. Myogenesis differentiated cells were fixed with 4% PFA for 10 min, permeabilized with 0.5% Triton X-100 in PBS for 10 min, stained with the primary antibody MY-32 (Abcam, Cat# ab51263) overnight at 4°C. The following procedures were the same as we described above. The antibody used in this study are shown in Supplementary Table S4.

### ChIP

For the ChIP (Chromatin Immunoprecipitation) assay, 200 drops on day 3.3 and 1 μl H3K27me3 antibody were enough for each ChIP assay. Cells were collected and crosslinked with 1% formaldehyde for 10 min at 37°C, then quenched by 0.125 M glycine. Cell pellets were lysed in SDS containing buffer (0.3% SDS; 50 mM Tris–HCl, pH 8.0; 20 mM EDTA) freshly supplied with protease inhibitors cocktail (Bimake, Cat# B14001) and sheared to 200–500 bp fragments by sonication. Fragmented chromatin was centrifuged at 13 000 rpm for 20 min at 4°C and the supernatants were diluted in dilution buffer (16.7 mM Tris–HCl, pH 8.0; 0.01% SDS; 1.1% Triton X-100; 1.2 mM EDTA; 167 mM NaCl). The chromatin complex was incubated for 6 h with H3K27me3 antibody (Cell Signaling Technology, Cat. No. 9733), then precipitated by 20 μl protein A/G beads (Smart-lifescience, Cat. No. SA032025) for 2 h rotating at 4°C. The precipitated chromatin was sequentially washed 3 times with RIPA buffer (50 mM HEPES, pH 8.0; 1% NP40; 0.7% DOC; 0.5 M LiCl) and then twice with TE buffer (50 mM Tris–HCl, pH 8.0; 1 mM EDTA). Precipitated chromatin complexes were eluted in elution buffer (50 mM Tris–HCl, pH 8.0; 1mM EDTA, 1% SDS) for 1 h at 65°C, and the supernatant was incubated at 65°C for 6 h on a thermos shaker for de-crosslinking.

RNA and proteins were digested using RNase A or proteinase K, respectively. And the DNA was purified by phenol chloroform extraction and ethanol precipitation. We eluted the samples with 30 μl water for ChIP-seq and qPCR analysis. The enrichment for ChIP-qPCR and ChIP-seq was normalized to the sample incubated without antibody. The primer sequences of qPCR and antibody are shown in Supplementary Table S1 and S4.

### Cleavage under targets & tagmentation (CUT & Tag)

Cleavage under targets & tag mentation (CUT & Tag) (42) were conducted in E14 mESCs to profile the chromatin association of hnRNPK. 1.0 million cells were collected for CUT & Tag assay of hnRNPK according to the manufacturer's protocol (Yeasen, Cat. No. 12598ES12). 2 μg hnRNPK antibody (Invitrogen, MA5-36291) was used for each CUT & Tag assay. The DNA library was prepared through PCR according to the manufacturer's protocol (Yeasen).

### ChIRP

ChIRP (Chromatin isolation by RNA purification) was performed as previously described with modifications (43), the 50–59-nt DNA biotinylated probes (BGI tech), more stringent crosslinking, hybridization and wash conditions were employed. About 5 × 10^7^ mESCs were crosslinked in 10-cm dish by 3% formaldehyde for 10 min at 37°C, quenched by 0.375 M glycine and harvested at 1500 rpm for 10 min at 4°C. Cells were resuspended in 800ul lysis buffer (50 mM Tris–Cl, pH 7.0; 10 mM EDTA; 1% SDS) with addition of protease inhibitor cocktail, PMSF and RNase inhibitor. The lysate was sonicated to 200–500 bp, then centrifuged at 13 000 rpm for 20 min at 4°C. We used 100 pmol probe mix for 800 μl lysis, and incubated the chromatin complex for 6 h at 37°C rotating all the time. The chromatin complex was then immunoprecipitated by streptavidin beads (Thermo, Cat. No. 20349) for 2 h at 37°C. The precipitated chromatin was sequentially washed 4 times by wash buffer (2x SSC; 0.5% SDS). DNA was eluted sequentially by RNase elution buffer (50 mM NaHCO_3_, 1% SDS, 100 μg RNaseA, 100 U RNase H) at 37°C for 1 h. De-crosslinked chromatin was subjected to proteinase treatment and the DNA was prepared as described in ChIP-seq method. The enrichment of ChIRP-seq signals were normalized to the *lncRNA-Smad7* knock-out cells. The sequences of probes used in this study are listed in Supplementary Table S3.

### ChIRP-MS

Mass spectrometry was performed followed ChIRP experiment. Equal amount of E14 cells were used for the ChIRP and control group. Proteins was eluted in 300 μl RIPA lysis buffer (Solarbio) with 10 μl RNaseA (20 mg/ml) and 10 μl RNaseH1 (10 U/μl) and incubated 2 h at 37°C. The beads were discarded by centrifugation, and the supernatant were recovered for the following experiment. 5× SDS loading buffer was added to the supernatant and the samples were prepared for 10 min at 100°C. Protein samples were separated in SDS-PAGE gel, and stained with Coomassie brilliant blue R-250. Each lane was cut into 2 pieces for the following mass spectrometry.

The gel bands of interest were excised from the gel, reduced with 5 mM of DTT and alkylated with 11 mM iodoacetamide which was followed by in-gel digestion with sequencing grade modified trypsin in 50 mM ammonium bicarbonate at 37°C overnight. The peptides were extracted twice with 0.1% trifluoroacetic acid in 50% acetonitrile aqueous solution for 1 h and then dried in a speedVac. Peptides were redissolved in 25 μl 0.1% trifluoroacetic acid, and 6μl of the extracted peptides were analyzed by Orbitrap Fusion mass spectrometer.

For LC–MS/MS analysis, the peptides were separated by a 120 min gradient elution at a flow rate 0.30 μl/min with a Thermo-Dionex Ultimate 3000 HPLC system, which was directly interfaced with an Orbitrap Fusion mass spectrometer (Thermo Fisher Scientific, Bremen, Germany). The analytical column was a home-made fused silica capillary column (75 μm ID, 150 mm length; Upchurch, Oak Harbor, WA) packed with C-18 resin (300 Å, 5 μm, Varian, Lexington, MA). Mobile phase A consisted of 0.1% formic acid, and mobile phase B consisted of 100% acetonitrile and 0.1% formic acid. The Q Exactive mass spectrometer was operated in the data-dependent acquisition mode using Xcalibur 2.1.2 software and there was a single full-scan mass spectrum in the orbitrap (300–1800 *m*/*z*, 70 000 resolution) followed by 20 data-dependent MS/MS scans at 27% normalized collision energy (HCD). The MS/MS spectra from each LC–MS/MS run were searched using an in-house Proteome Discoverer (Version PD1.4, Thermo-Fisher Scientific, USA). Static peptide modification included carbamidomethylation (C), dynamic oxidation (M). Two trypsin missed cleavage was allowed. Precursor tolerance and ion fragment tolerance were set at 20 ppm and 0.02 Da, respectively. Confidence levels were set to 1% FDR (high confidence) and 5% FDR (middle confidence). The top ranked proteins of ChIRP-MS were listed in Supplementary Table S6.

### Dual-luciferase reporter assay

*Bmp2* promoter region (chr2: 133551243–133551936) with or without GT-repeats and *Pou5f1* promoter (chr17: 35503947–35504243) region were cloned into pGL3-promoter vector. mESCs E14 (1 × 10^5^ in each well of 12-well plate) were co-transfected by 900 ng luciferase reporter vector and 100 ng renilla vector (internal control) with lipofectamine 2000. Cells were harvested 48 h after transfection. Luciferase activities were examined using the Dual-Luciferase Reporter Assay System (Promega Cat# E1910) and measured by enzyme-labeled instrument (PerkinElmer).

### ELISA

The autocrine BMP2 protein levels were quantified using a commercially available ELISA kit (Abcam, Cat. No. ab119582). All samples were collected from the media culturing cells for 48 h. The media were centrifuged 1000 rpm for 5 min and measured immediately or stored at −80°C less than one month. All samples were assayed according to the manufacturer's instructions and tested in duplicates by personnel blinded for each group. The optical density of each well was determined using a microplate reader at an absorbance of 450 nm. No interference and no cross-reactivity were expected based on the manufacturer's instructions. The minimum detectable dose (MDD) of BMP2 ELISA ranged from 50 to 200 pg/ml. The dynamic range of BMP2 ELISA ranged from 15.625 to 1000 pg/ml.

### Protein production and purification

The recombinant Flag-tagged hnRNPK of mouse was constructed in PCI backbone. The plasmid was transfected into the HEK293T cells when the optical density of the culture reached 0.7 (20 μg plasmid for each T75 flask cells). Each T75 flask cells were passaged to one 15 cm dish 12 h post-transfection and harvested 36 h post-transfection. The cell pellet of each dish was suspended in 1 ml cold lysis buffer (400 mM KCl; 20 mM MgCl_2_; 40 mM HEPES–KOH, PH 7.0; 0.5% NP40), containing 1 mM DTT and EDTA-free protease inhibitor cocktail. Cells were lysed by sonication on ice for 30 s intermittently at 30% power. The resulting lysates were centrifuged at 4°C for 15 min at 13 000 rpm, then the supernatants were saved for purification experiments. The 50 μl anti-Flag resins were added to the supernatant of each sample and incubated for 6 h at 4°C. Then, the resins were washed with lysis buffer for four times at 4°C followed with incubation with the elution buffer (20 mM Tris–HCl, PH 7.6; 500 mM NaCl) containing 300 μg/ml flag peptide for twice (125 μl each time) at 4°C. The hnRNPK protein was further purified with heparin column and gel-filtration column (HiLoad 16/60 Superdex200) by AKTA system (GE Healthcare). The protein purity was monitored by SDS-PAGE.

### IVT (*in vitro* transcription)

Full length, (CA)_12_, and (UG)_25_ containing segments of *lncRNA-Smad7* were transcribed *in vitro*. Briefly, the purified DNA templates of *lncRNA-Smad7* fragment were transcribed *in vitro* by T7 polymerase (0.2 mg/ml) for 3 h at 37°C. DNase I was then added to the transcribed RNA for 30 min at 37°C. The mixture was centrifuged to remove any precipitate. The digested RNA was purified by phenol–chloroform (PH 5.2) at the ration 1:1 followed with centrifugation for 30 min at 13 000 rpm at 4°C. The supernatant was concentrated by ultrafiltration spin columns (Millipore) with DEPC water for 100 000 folds at least. Finally, the RNA was concentrated to ∼0.5–2.0 mg/μl.

### EMSA (electrophoretic mobility shift assay)

For RNA–protein EMSA: Increasing amounts of purified hnRNPK was incubated with 1.5 pmol in vitro transcribed *lncRNA-Smad7* fragments for 30 min at RT (10 mM Hepes PH 7.5; 400 mM NaCl; 5 mM MgCl_2_; 0.01% Triton X-100; 10% glycine; 2 mM DTT).

For RNA-DNA EMSA: The Cyanine5 (Cy5) labeled single strand oligonucleotides were generated by incubating 5’NH2 labeled oligonucleotides (Sangon Biotech) and the reactive dye Cyanine5 NHS ester (LumiProbe) at 1:1 molar ratio and kept at RT for 4 h. Labeled nucleic acid were then separated from excess free fluorophores through ethanol precipitation. Increasing amounts of the *in vitro* transcribed *lncRNA-Smad7* fragments were incubated with 0.15 pmol Cy5 labeled single strand oligonucleotide [(TG)_17_], [(CA)_17_], and [TCTAGTGA-(TG)_17_-TCCATGTG] for 30 min at RT. Double strands 50nt oligonucleotides (CA:TG)_17_ were annealed by Cy5 labeled [TCTAGTGA-(TG)_17_-TCCATGTG] and [CACATGGA-(CA)_17_-TCACTAGA] oligonucleotide.

For RNA-DNA and R-loop antibody S9.6 EMSA: the RNA/DNA hybrids containing 0.15 pmol 5′-cy5–labeled DNA and 1.8 pmol RNA (12 folds to the DNA), and increasing amount of S9.6 antibody were incubated in the buffer [10 mM Tris–HCl (pH 7.5), 2.5mM MgCl_2_, 2.5M Glycerol, 50 mM NaCl and 1 mM DTT] at RT for 30 min. Then, 2.5 U and 5.0 U RNase H1 (NEB), 0.01mg RNase A (Transgene) or vehicle was added to the DNA/RNA/S9.6 complex.

The complexes were separated in a 1.2% agarose gel in 0.5× TAE buffer at 100 V for 50 min on ice. Finally, fluorescent signal was capture by Amersham Typhoon (GE Healthcare Bio-Sciences AB, 67130069) and nucleic acids stained with Superstain (CWBIO).

### Negative staining EM of RNA

The structure of *lncRNA-Smad7* was verified by negative staining electron microscopy (EM). All RNA samples were diluted at a final concentration of 2.5 μM in the DEPC water and negatively stained in 2% (w/v) uranyl acetate solution following the standard deep stain procedure on holey-carbon coated EM copper grids covered with a thin layer of continuous carbon (44). And all the negatively stained specimens were examined on an FEI Tecnai-F20 electron microscope operated at 200 kV acceleration voltage at 50 000 nominal magnification with a range of defocus from 3 to 3.5 μm. The electron micrographs were taken on a Gatan Ultrascan4000 4k × 4k CCD camera.

### DRIP-qPCR

DNA–RNA immunoprecipitation (DRIP) was performed by using S9.6 antibody recognizing DNA-RNA hybrid along chromosomes as described previously (45–47) with several modifications. In brief, 2 × 10^7^ cells were collected and the genome was extracted by the genome extraction kit (TIANGEN).

RNase A and RNase H treatment: half of the genomic DNA (gDNA) was treated by 5 μl RNase A for 2 h at 37°C, then 1/10 volume of 10× RNase H buffer and RNase H (NEB) was added, followed by incubation at 37°C overnight. RNase-treated and -untreated gDNAs were digested with Mse I, Dde I, Alu I, and Mbo I (NEB; final concentration: 75 U/ml for each enzyme) at 37°C. Fragmented gDNA was purified by phenol:chloroform (25:24), resuspended in TE buffer and quantified by Qubit 3.0 (Invitrogen). Then, each sample was immunoprecipitated with 1× DRIP binding buffer [10 mM NaPO4 (pH 7.0), 140 mM NaCl and 0.05% Triton X-100] and S9.6 antibody (American Type Culture Collection, HB-8730, 10 μg for 1 μg gDNA) in a shaker at 4°C overnight. After adding Protein A/G beads and incubating at 4°C for 3 h, the beads were washed four times with 1× DRIP binding buffer. Next, the S9.6 associated complexes were eluted by elution buffer [50 mM Tris–HCl (pH 8.0), 0.5% SDS and 10 mM EDTA] containing proteinase K at 55°C for 1 h. The complex was purified with phenol/chloroform extraction, and the supernatant was transferred into a new tube. 1/10 volume of 3 M NaAc, 1 μl Acryl carrier (Solarbio), and 2.5 volume ethanol was added to precipitated the complex at −20°C at least 2 h. The DRIPed DNA pellet was washed with 70% ethanol, air-dried, resuspended in DEPC water and used for qPCR. The sequence of the primers are listed in Supplementary Table S1.

### RNA-seq analysis

Cells were collected by trypsin digestion and the RNA was isolated by RNA isolation kit (Dakewe, Cat. No. 8034111). High-throughput sequencing was performed by Novogene on a Hiseq X. We mapped the RNA-seq data to the mouse reference genome (mm10) using HISAT2 (version 2.1.0). The levels of gene expression were calculated by Cufflinks (version 2.2.1) based on mm10 annotations. Differential testing and log_2_ fold change calculations were performed using Cuffdiff (version 2.2.1), with the implementation of two biological replicates. Heatmap was performed by Rstudio and Gene Ontology analysis was performed using DAVID (https://david.ncifcrf.gov/).

### Statistical analysis

The intensity of the western blotting bands was quantified by Image J and presented after normalization to the loading control. β-Actin (Actb) was used as the internal control for qPCR analysis. Quantitative data in this study are presented as mean ± S.D. from three independent experiments at least (*n* ≥ 3) unless otherwise stated, and compared statistically by unpaired Student's *t* test. Statistical significance was indicated as follows: *P ≤ 0.05, **P ≤ 0.01, ***P ≤ 0.001. Statistical parameters for each experiment, including values of *n* and statistical significance, can be found in the figure legends. Graphs were generated using GraphPad Prism (6.01).

## RESULTS

### Nodal-driven SMAD signaling activates *lncRNA-Smad7* transcription in mESCs

**Figure 1.**
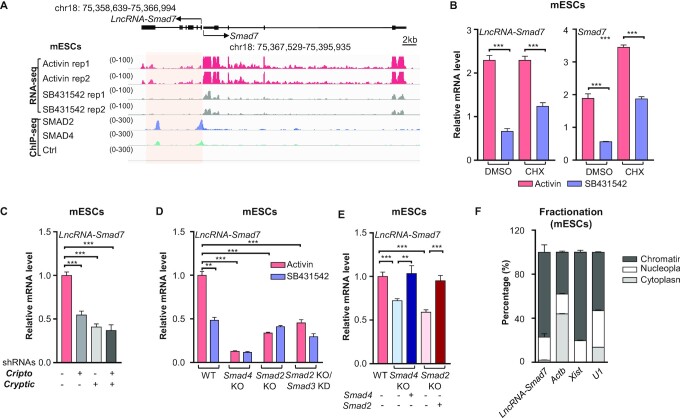
Nodal-driven SMAD signaling activates the transcription of *lncRNA-Smad7* in mESCs. (**A**) Gene tracks of RNA-seq data for Activin A (hereafter, Activin)- and SB431542-treated mESCs (GSE115169); ChIP-seq of SMAD2 and SMAD4 (GSE125116) in mESCs at *lncRNA-Smad7* and *Smad7* loci (mm10: mouse reference genome 10). (**B**) qPCR analysis of *lncRNA-Smad7* and *Smad7* expressions in Activin (50 ng/ml)- and SB431542 (10 μM)- treated mESCs for 2 h pretreated with cycloheximide (hereafter, CHX, 0.1 mg/ml) or DMSO for 1 hour. DMSO was used as the negative control for CHX. (**C**) qPCR analysis of *lncRNA-Smad7* expression in *Cripto* KD, *Cryptic* KD, and *Cripto*/ *Cryptic* double KD mESCs by shRNAs. KD, knock-down. (**D**) qPCR analysis of *lncRNA-Smad7* expression in the indicated mESCs with Activin (50 ng/ml) and SB431542 (10 μM) treatment. KO, knock-out. (**E**) qPCR analysis of *lncRNA-Smad7* expression in *Smad2* KO and *Smad4* KO mESCs with or without expression of *Smad2* or *Smad4* respectively. (**F**) Fractionation assay showed the subcellular localization of *lncRNA-Smad7* and the other indicated transcripts in mESCs. The *Actb* (*β-actin*), *U1*, and *Xist* mRNAs were used as cytoplasm, nucleoplasm, and chromatin markers for the fractions respectively. All RNA abundance values are absolutely quantified, the data are representative of three independent experiments. (B–E) All data are representative of three independent experiments. Data are presented as mean ± S.D. (Standard Deviation), *n* = 3, asterisks indicate a difference from control, *P < 0.05, **P < 0.01, ***P < 0.001 (two-tailed Student's *t* test). See also Supplementary Figure S1.

We set out to explore lncRNAs responsive to Nodal signaling in mESCs (Supplementary Figure S1A). Activin A (hereafter, Activin) is used as a substitute for Nodal in this study because it is more readily available from mammalian sources than Nodal and they act through the same receptors, except that Nodal also requires the Cripto and Cryptic co-receptors (48,49). We used Activin to activate Nodal signaling and the ALK4/5/7 inhibitor SB431542 to block stimulation by Nodal-like factors in mESCs. RNA-seq analysis showed that transcription of both *lncRNA-Smad7* (Supplementary Figure S1B) and *Smad7* are induced by Activin (Figure 1A, B). Copy number quantification indicated that *lncRNA-Smad7* transcripts were present at about two copies per cell in both E14 and C2C12 cells (Supplementary Figure S1C). ChIP-seq analysis of SMAD2 and SMAD4 indicated that these transcription factors bind at the promoter and gene body of *lncRNA-Smad7* in mESCs (Figure 1A). In addition, *lncRNA-Smad7* expression was significantly decreased in mESCs with knockdown for the Nodal co-receptors Cripto and Cryptic (Figure 1C and Supplementary Figure S1D), which suggested that *lncRNA-Smad7* is indeed responsive to Nodal signaling.

To verify that *lncRNA-Smad7* is a primary target of Nodal signaling, mESCs were pretreated with the protein synthesis inhibitor cycloheximide (hereafter, CHX) and *lncRNA-Smad7* and *Smad7* expression were quantified by qPCR. The results indicated that both *lncRNA-Smad7* and *Smad7* are transcriptionally activated in the presence of Activin, suggesting that their expression did not require nascent synthesis of protein factors, and that *lncRNA-Smad7* is a primary target of Nodal signaling (Figure 1B). Moreover, no induction of *lncRNA-Smad7* was observed upon Activin treatment in mESCs with *Smad2* KO, *Smad2* KO*/Smad3* KD or *Smad4* KO (Figure 1D), while the induction of *lncRNA-Smad7* was rescued by reconstituting *Smad2* or *Smad4* expression (Figure 1E). Taken together, these results demonstrated that *lncRNA-Smad7* is directly activated by Nodal-driven SMAD signaling in mESCs.

Fractionation assays to determine *lncRNA-Smad7* localization indicated that it mainly resides in the nucleus of mESCs, supporting its function in associating with chromatin (Figure 1F). Moreover, qPCR assays indicated that *lncRNA-Smad7* transcripts were highly enriched in skeletal muscle and heart on postnatal day 14 or week 8 in adult mice (Supplementary Figure S1E and S1F), and was expressed at lower levels in the liver, heart, and brain of embryos (Supplementary Figure S1E and S1F). It should be noted that no obvious homolog of murine *lncRNA-Smad7* was identified in other mammals (Supplementary Figure S1G). We then cloned *lncRNA-Smad7* transcripts from mESCs by RACE (rapid amplification of cDNA ends), which revealed an alternative isoform that was 81 bp shorter at the 5’ end of exon1 compared with other previously reported transcript isoforms (37,38) (Supplementary Figure S1H).

### *LncRNA-Smad7* knockdown impairs cardiomyocyte differentiation in mESCs

**Figure 2.**
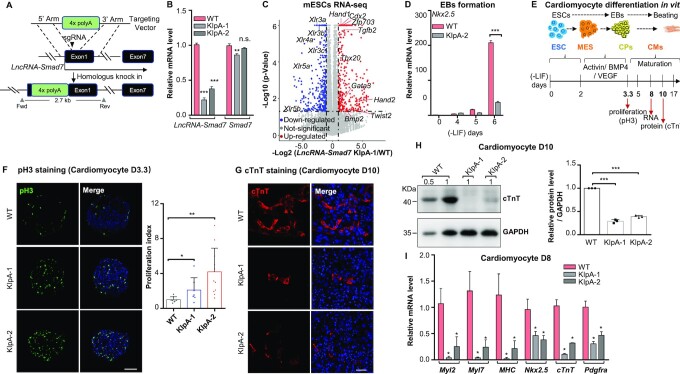
Knock-down of *lncRNA-Smad7* impairs cardiomyocyte differentiation of mESCs. (**A**) Scheme of the strategy to generate 4× polyA stop cassette knock-in mESCs at 5’ end of *lncRNA-Smad7*. (**B**) qPCR analysis of *lncRNA-Smad7* and *Smad7* expressions of the *lncRNA-Smad7* KIpA and wide type (WT) mESCs. (**C**) Volcano plot of the DEGs (2-fold cutoff) in *lncRNA-Smad7* KIpA cells compared with WT mESCs. The data are representative of two independent experiments. (**D**) qPCR analysis of *Nkx2.5* expression in *lncRNA-Smad7* KIpA and WT EBs on the indicated day. (**E**) Schematic drawing showed in vitro differentiation of mESCs to cardiomyocytes. MES, mesoderm stage; CPs, cardiac progenitors; CMs, cardiomyocytes. (**F**) (Left) Immunofluorescence staining showed the pH3 (green) in *lncRNA-Smad7* KIpA and WT cells on CM day 3.3 (CM D3.3). Nuclei were stained with DAPI (blue); scale bar, 100 μm; (right) quantification of pH3 positive ratios showed in (F, left). Scatter plots represent the individual views (normalized to the WT sample). Data are presented as mean ± S.D., two-tailed Student's *t* test, *n* ≥ 7, *P < 0.05, **P < 0.01. (**G**) Immunofluorescence staining of cTnT (red) for *lncRNA-Smad7* KIpA and WT CMs on day 10. Nuclei were stained with DAPI (blue); scale bar, 25 μm. (**H**, left) Western blot analysis of the total cTnT protein levels in *lncRNA-Smad7* KIpA and WT CMs on day 10 (D10), GAPDH was used as the loading control. 0.5 and 1 indicated the percentage of loading for each sample; (right) quantification of the cTnT protein levels showed in (H, left) (normalized to WT samples of each independent experiments). Scatter plots represent the three biologically independent experiments. The intensities for each samples were measured by ImageJ. (**I**) qPCR analysis of cardiac-specific genes on day 8 (D8) in *lncRNA-Smad7* KIpA and WT CMs. (B, D, H, I) All data are representative of three independent experiments. Data are presented as mean ± S.D., *n* = 3, *P < 0.05, **P < 0.01 (two-tailed Student's *t* test). See also Supplementary Figures S2 and S3.

We next used CRISPR-Cas9 to generate *lncRNA-Smad7* knockdown (KD) cell lines (*lncRNA-Smad7* KIpA) by inserting a 4× polyA transcription stop cassette in exon1 (Figure 2A and Supplementary Figure S2A). QPCR analysis showed a ∼70% reduction in *lncRNA-Smad7* expression in the *lncRNA-Smad7* KIpA cells, whereas no major effects were observed on *Smad7* expression (Figure 2B). Further RNA-seq analysis in *lncRNA-Smad7* KIpA and WT mESCs with gene ontology (GO) analysis showed that the differentially expressed genes (DEGs) were mainly involved in development (Supplementary Figure S2B). However, *lncRNA-Smad7* KIpA mESCs exhibited morphology and alkaline phosphatase (AP) activity similar to WT mESCs (Supplementary Figure S2C). Using a 2-fold cutoff to identify up- and down-regulated DEGs in the *lncRNA-Smad7* KIpA mESCs revealed a set of up-regulated DEGs well-known to participate in heart development and trophoblast lineage determination, including *Bmp2*, *Cdx2* and *Hand1*, etc. (Figure 2C and Supplementary Figure S2D). In addition, BMP2 is a known factor regulating *Hand1* and *Cdx2* expression during early development (50–52).

We then induced embryoid body (EB) formation for mesendoderm and ectoderm differentiation assays in WT and *lncRNA-Smad7* KIpA mESCs to investigate the role(s) of *lncRNA-Smad7* in these differentiation processes (Supplementary Figure S2E) (53,54). Compared to WT cells at day 0, the expression levels of both *lncRNA-Smad7* and *Smad7* were transiently reduced (i.e. lower on EB day 4 and ectodermal differentiation day 3) (Supplementary Figure S2F, G) during the mesendoderm differentiation. While depletion of *lncRNA-Smad7* during differentiation did not lead to consistent effects on *Smad7* expression (Supplementary Figure S2H). No obvious different effect was detected during the ectoderm-specific gene expression *in lncRNA-Smad7* KD cells (Supplementary Figure S2I), but early mesendoderm differentiation was significantly impacted (Supplementary Figure S2J) compared to wild type cells. Furthermore, expression of the cardiomyocyte marker *Nkx2.5* was dramatically decreased in *lncRNA-Smad7* KIpA EBs (Figure 2D), suggesting that *lncRNA-Smad7* might participate in cardiomyocyte differentiation.

We next investigated the functional impacts of *lncRNA-Smad7* in cardiomyocyte differentiation (Figure 2E) (19,55). Successful cardiomyocyte differentiation was validated through classic immunofluorescence staining assays for cTnT (cardiac troponin T) (Supplementary Figure S3A). The cardiomyocyte markers *Nkx2.5* and *cTnT* were strongly induced in cardiomyocytes (CMs), whereas *lncRNA-Smad7* expression was reduced on day 3.3 (80 h) of cardiac progenitor cell (CPs) differentiation (Supplementary Figure S3B). Changes in cardiac progenitor cell proliferation rates are known to lead to defects in cardiomyocyte maturation (13,23). It is thus notable that CP proliferation rates in *lncRNA-Smad7* KIpA EBs increased significantly, as indicated by phospho-Histone H3 (pH3) levels at this time point (Figure 2F). However, no obvious difference was observed in EB size between WT and *lncRNA-Smad7* KIpA cells on cardiomyocyte day 3.3 (CM D3.3) (Supplementary Figure S3C).

Interestingly, immunofluorescence staining showed that the proportion of cTnT positive CMs was significantly reduced among *lncRNA-Smad7* KIpA cells compared to WT cells (Figure 2G). Consistent with this finding, cTnT protein levels were significantly decreased in *lncRNA-Smad7* KIpA CMs on day 10 (Figure 2H). Moreover, the expression of cardiac-specific genes (*e.g*. *Myl2*, *Myl7*, *MHC*, *Nkx2.5* and *cTnT*) was markedly reduced in *lncRNA-Smad7* KD cells on day 8 of cardiomyocyte differentiation (Figure 2I). Thus, reducing *lncRNA-Smad7* expression can stimulate the proliferation of cardiac progenitor cells but impair cardiomyocyte maturation in vitro.

### *LncRNA-Smad7* represses *Bmp2* expression and associates at its promoter

**Figure 3.**
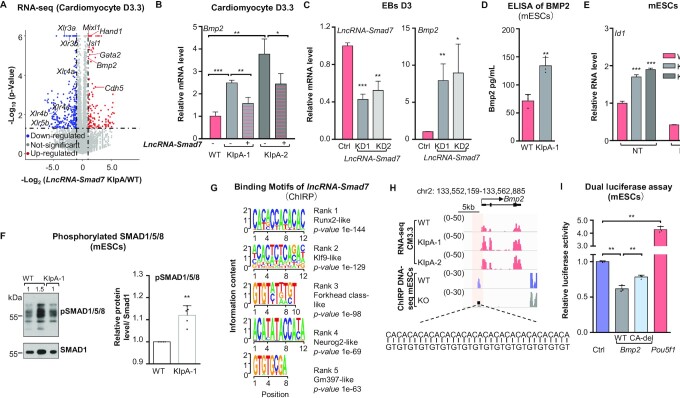
*LncRNA-Smad7* represses *Bmp2* transcription during cardiomyocyte differentiation and binds at *Bmp2* promoter. (**A**) Volcano plot of the DEGs (2-fold cutoff) in *lncRNA-Smad7* KIpA cells compared with WT cells in the stage of cardiac progenitor cells (cardiomyocyte D3.3). The data are representative of two independent experiments. (**B**) qPCR analysis of *Bmp2* expression in the indicated cells on cardiomyocyte D3.3. (**C**) qPCR analysis of *Bmp2* and *lncRNA-Smad7* expression in *lncRNA-Smad7* KD and control EBs on day 3 (D3). (**D**) ELISA assay showed autocrine Bmp2 protein levels in *lncRNA-Smad*7 KIpA mESCs compared to WT. The data are representative of three biologically independent experiments. (**E**) qPCR analysis of *Id1* expression in *lncRNA-Smad7* KIpA and WT mESCs. BMP type I receptor inhibitor LDN193189 (hereafter, LDN, 1 μM) was used to block BMP signaling. (**F**) Western blot analysis (left panels) and quantification (right panel) of phospho-Smad1/5/8 in *lncRNA-Smad7* KIpA and WT mESCs (normalized to WT samples of each independent experiments). Smad1 was used as the loading control. Data are presented as mean ± S.D. Scatter plots represent the biologically independent experiments (*n* = 5). 1 and 1.5 indicated the amount of loading for each sample. (**G**) Motif analysis of the ChIRP DNA-seq of *lncRNA-Smad7*. (**H**) IGV tracks showed: RNA-seq of WT and *lncRNA-Smad7* KIpA cells at CM3.3; DNA-seq of *lncRNA-Smad7* ChIRP in WT and *lncRNA-Smad7* whole locus knock-out (KO) mESCs at *Bmp2* locus (mm10). (**I**) Dual-luciferase reporter assay showed the activities of *Bmp2* and *Pou5f1* promoter regions in mESCs. Ctrl was the empty vector; CA-del, *Bmp2* promoter region without the (CA:TG)_17_-repeats. (B–E, I) All data are representative of three independent experiments. Data are presented as mean ± S.D., *n* = 3, *P < 0.05, **P < 0.01, ***P < 0.001 (two-tailed Student's *t* test). See also Supplementary Figure S4.

Next, we performed RNA-seq analysis in both *lncRNA-Smad7* KIpA and WT cardiac progenitor cells. GO analysis showed that the up-regulated DEGs in *lncRNA-Smad7* KIpA cells (*n* = 101) were mainly associated with organismal development, including heart development (Supplementary Figure S4A). In line with this finding, a large suite of heart development-related genes were significantly up-regulated in *lncRNA-Smad7* KIpA cells on CM D3.3 (Figure 3A). In particular, the expression of cardiovascular-specific genes such as *Bmp2*, *Hand1* and *Hand2* were also dramatically increased in *lncRNA-Smad7* KIpA cells on CM D3.3 (Figure 3A), which were also significantly up-regulated in the *lncRNA-Smad7* KIpA mESCs (Figure 2C).

We then introduced a *lncRNA-Smad7* expression vector into *lncRNA-Smad7* KIpA mESCs to overexpress *lncRNA-Smad7*. The elevated expression of *Bmp2* was repressed under *lncRNA-Smad7* overexpression in *lncRNA-Smad7* KIpA cells (Figure 3B), suggesting that *Bmp2* was directly suppressed by *lncRNA-Smad7*. We further generated two *lncRNA-Smad7* KD mESC lines mediated by shRNAs and initiated differentiation in both WT and KD ESCs. Consistent with the KIpA phenotype, *Bmp2* expression was up-regulated in *lncRNA-Smad7* KD cells (Figure 3C). In addition, the highest levels of *lncRNA-Smad7* expression were detected in embryonic mouse hearts at E10.5 to E16.5, whereas *Bmp2* expression concomitantly declined during this period (Supplementary Figure S4B). Conversely, in the early stages of cardiomyocyte differentiation in vitro, *Bmp2* expression increased while *lncRNA-Smad7* expression decreased (Supplementary Figure S3B).

In addition, we quantified autocrine BMP2 levels in culture media by ELISA, which showed that secreted BMP2 levels were higher in *lncRNA-Smad7* KIpA cells than that in WT cells from 71.4 to 134.1 ng/ml (Figure 3D). In line with this result, expression of the downstream BMP target, *Id1*, was increased in *lncRNA-Smad7* KIpA cells compared to that in WT mESCs (Figure 3E). We also detected a slight but steady ∼1.1 fold increase in phospho-SMAD1/5/8 at SMAD1/5 residues Ser463/465 and Smad8 sites Ser465/467 in *lncRNA-Smad7* KIpA mESCs, further illustrating that *lncRNA-Smad7* KD leads to upregulation of BMP signaling (Figure 3F).

We then performed ChIRP-seq (chromatin isolation by RNA purification and sequencing) (43) for endogenous *lncRNA-Smad7* using antisense DNA oligos tiling along the entire transcript of *lncRNA-Smad7* to obtain genome-wide binding sites of *lncRNA-Smad7*. To this end, we used *lncRNA-Smad7* truncation knock-out mutant (KO) mESCs as the negative control. We found that *lncRNA-Smad7* strongly associated with its own gene locus in WT mESCs but not in KO cells, validating the specificity of DNA affinity capture (Supplementary Figure S4C). Interestingly, motif analysis of ChIRP DNA-seq data indicated that *lncRNA-Smad7* preferentially binds at CA- or GT-rich genomic regions (Figure 3G). Moreover, the ChIRP DNA-seq results showed that *lncRNA-Smad7* could bind a *Bmp2* promoter region containing (CA:TG)_17_-repeats (Figure 3H).

We then cloned the *Bmp2* promoter region harboring the (CA:TG)_17_-repeats into the pGL3-promoter vector to examine its transcriptional regulatory activity. The *Pou5f1* promoter was used as a positive regulatory element control. Luciferase reporter assays in mESCs showed that the *Bmp2* promoter region exerted repressive regulatory effects in a manner partially dependent on (CA:TG)_17_-repeats, since deletion of the (CA:TG)_17_-repeat region led to a significant reduction in transcriptional repression of luciferase signal (Figure 3I).

Collectively, these results indicated that *lncRNA-Smad7* represses *Bmp2* expression in both mESCs and cardiac progenitor cells (CM D3.3) and binds to a promoter region containing (CA:TG)_17_-repeats.

### *LncRNA-Smad7* directly associates with *Bmp2* promoter via (CA)12-repeats *in vitro*

**Figure 4.**
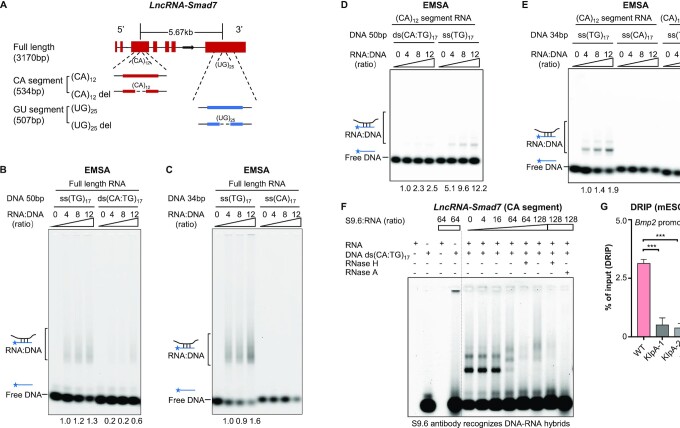
*LncRNA-Smad7* directly binds at the promoter region of *Bmp2* via CA repeats *in vitro*. (**A**) Schematic drawing showed in vitro transcription of full length, (CA)_12_ and (UG)_25_-containing segments of *lncRNA-Smad7*. (**B**) EMSA showed the binding affinity of 50 bp single strand TG-repeats ss(TG)_17_ and double strands (CA:TG) repeats ds(CA:TG)_17_ with the increasing amounts of the full length of *lncRNA-Smad7*. (**C**) EMSA showed the binding affinity of 34 bp ss(TG)_17_ and ss(CA)_17_ with the increasing amounts of the full length of *lncRNA-Smad7*. (**D**) EMSA showed binding affinity of 50 bp ds(CA:TG)_17_ and ss(TG)_17_ with the increasing amounts of the *in vitro* transcribed (CA)12 containing segment of *lncRNA-Smad7*. (**E**) EMSA showed binding affinity of 34 bp ss(TG)_17_ or ss(CA)_17_ repeats with the increasing amounts of (CA)_12_ containing segment of *lncRNA-Smad7* and (CA)_12_ containing segment without (CA)_12_ repeats. 0.15 pmol Cy5-labeled DNA was used as a fixed amount in (B–E). (**F**) EMSA showed binding affinity of R-loop antibody S9.6 and DNA/RNA complex, 2.5 U and 5 U of RNaseH1 or 0.1 mg RNaseA were added in the indicated samples. 0.15 pmol 5′-cy5–labeled DNA and 1.8 pmol RNA (12-fold to the DNA) were used as a fixed amount in this experiment. (**G**) DRIP-qPCR showed the R-loop signal at the *Bmp2* promoter region in WT and *lncRNA-Smad7* KIpA mESCs. RNase indicated RNase A and RNase H1. (G) All data are representative of three independent experiments. Data are presented as mean ± S.D., *n* = 3, ***P < 0.001 (two-tailed Student's *t* test). See also Supplementary Figure S4.

To further characterize the binding interactions between *lncRNA-Smad7* and the promoter region of *Bmp2*, we conducted gel-shift assays using variants of *lncRNA-Smad7* transcribed *in vitro* (Figure 4A and Supplementary Figure S4D) with Cyanine 5 (Cy5)-labeled double stranded (ds) or single stranded (ss) DNA fragments containing (CA:TG)_17_-repeats. The results showed that full length *lncRNA-Smad7* associates with both ds and ss DNAs containing (TG)_17_-repeats, and that *lncRNA-Smad7* exhibited a greater shift with ss DNA than ds DNA (Figure 4B). In addition, *lncRNA-Smad7* could bind with ss DNAs containing (TG)_17_ repeats, but not with (CA)_17_-containing ss DNAs (Figure 4C). It should also be noted that both (CA)_12_- and (UG)_25_-repeats are present in *lncRNA-Smad7* transcripts (Figure 4A).

Next, we examined the DNA binding ability of *lncRNA-Smad7* RNA segments containing (CA)_12_- and (UG)_25_-repeats (Figure 4A). These assays indicated that the (CA)_12_-containing RNA segment (534 bp) could bind with either (TG)_17_-containing ss DNAs or (CA:TG)_17_-containing ds DNAs in a similar manner to full length *lncRNA-Smad7* (Figure 4D). In addition, its association with ss (TG)_17_ DNA was dependent on the (CA)_12_ sequence in the RNA (Figure 4E). In the contrast, the (CA)_12_-containing RNA segment showed negligible binding with ss (CA)_17_ DNA (Figure 4E). Negative staining EM (electron microscopy) of RNA revealed that deletion of the (CA)_12_ repeats dramatically impacted the in vitro folding conformation of the (CA)_12_-containing *lncRNA-Smad7* segment (Supplementary Figure S4E). Although the full length RNA did not associate with ss (CA)_17_ DNA, we detected interactions between the (UG)_25_ RNA segment with ss (CA)_17_ DNA (Supplementary Figure S4F), although it remains unclear why UG-repeats in full length *lncRNA-Smad7* cannot base pair with CA-repeats in ss DNA. We speculated that full length *lncRNA-Smad7* might form tertiary structures that allow RNA association with certain sequences (i.e. (TG)_17_ repeats) while inhibiting RNA binding with other sequences (i.e. (CA)_17_ repeats), which could be critical for its function suppressing *Bmp2* expression. We further analyzed the CA-repeats containing lncRNAs responsive to nodal/TGF-β signaling in mESCs (56) and hESCs (57), indicating that there may be CA-repeats containing lncRNAs which function similarly to mouse *lncRNA-Smad7* in both Mus Musculus and Homo Sapiens (Supplementary Table S5).

Previous reports have shown that DNA and RNA can form R-loops or triplexes to regulate gene expression (58–62). Since *lncRNA-Smad7* preferentially associates with the ss (TG)_17_ repeats over interactions with ds (CA:TG)_17_ of the *Bmp2* promoter in vitro, we hypothesized that *lncRNA-Smad7* may form an R-loop with DNA fragments containing (TG) repeats at the *Bmp2* promoter. Exposure to the R-loop antibody S9.6 in EMSAs resulted in a so-called supershift in DNA/RNA complex, while RNaseH1 treatment abolished the supershift band (Figure 4F), suggesting that the (CA)_12_-containing *lncRNA-Smad7* segment indeed forms an R-loop with DNA. Moreover, DRIP (DNA/RNA hybrid immunoprecipitation)-seq analysis in iPSCs (47) facilitated identification of an R-loop in the *Bmp2* promoter region that overlapped with the ChIRP signal of *lncRNA-Smad7* in mESCs (Supplementary Figure S4G). In light of this evidence, we performed DRIP-qPCR experiments using S9.6 antibody to validate the RNA/DNA hybrids (R-loop) at the *Bmp2* promoter in WT and *lncRNA-Smad7* KD mESCs. The DRIP-qPCR results showed that the R-loop signal was markedly stronger in WT mESCs than in *lncRNA-Smad7* KIpA cells (Figure 4G), indicating that *lncRNA-Smad7* forms the R-loop at the *Bmp2* promoter region. Taken together, these findings implied that *Bmp2* promoter R-loop could potentially contribute to the transcriptional repression of *Bmp2* by *lncRNA-Smad7*.

### Bmp2 KD rescues defects in cardiomyocyte differentiation in *lncRNA-Smad7* KD cells

**Figure 5.**
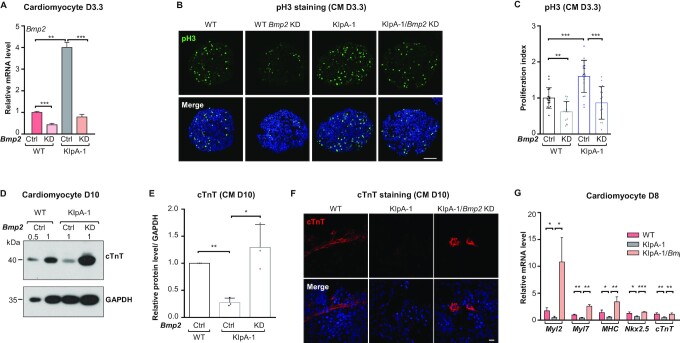
*Bmp2* KD rescues cardiomyocyte differentiation defects in *lncRNA-Smad7* KD cells. (**A**) qPCR analysis of *Bmp2* expression on CM D3.3. (**B**) Immunofluorescence staining showed the pH3 levels (green) in *Bmp2* KD, *lncRNA-Smad7* KIpA, *lncRNA-Smad7* KIpA/*Bmp2* KD and control EBs on CM D3.3. Nuclei were stained with DAPI (blue); scale bar, 100 μm. (**C**) Quantification of pH3 positive ratios showed in (B). Scatter plots represent the individual views (normalized to the WT sample). Data are presented as mean ± S.D., two-tailed Student's *t* test, *n* ≥ 14, **P < 0.01, ***P < 0.001. (**D**) Western blot showed the total protein level of cTnT in *lncRNA-Smad7* KIpA, *lncRNA-Smad7* KIpA/*Bmp2* KD and control CMs on D10. GAPDH was used as the loading control. 0.5 and 1 indicated the percentage of loading for each sample. (**E**) Quantification of the cTnT protein intensities showed in (D). Scatter plots represent the three biologically independent experiments. The intensities for each sample were measured by ImageJ. (**F**) Immunofluorescence staining of cTnT (red) for *lncRNA-Smad7* KIpA, *lncRNA-Smad7* KIpA/*Bmp2* KD, and WT CMs on D10. Nuclei were stained with DAPI (blue); scale bar, 25 μm. (**G**) qPCR analysis of key cardiac-specific genes in the indicated CMs on D10. (A, E, G) All data are representative of three independent experiments. Data are presented as mean ± S.D., *n* = 3, *P < 0.05, **P < 0.01, ***P < 0.001 (two-tailed Student's *t* test). See also Supplementary Figure S5.

Previous studies have shown that *Bmp2* or *Hand1* over-expression increases the proliferation rate of cardiac progenitor cells and consequently impairs cardiomyocyte maturation (13,23). Based on the results above, we assumed that up-regulation of cardiac-specific genes such as *Bmp2* and *Hand1* on CM D3.3 could impair cardiomyocyte differentiation. To test this possibility, we generated *Bmp2* KD mESCs by shRNA to verify whether defects in cardiomyocyte differentiation caused by *lncRNA-Smad7* depletion could be rescued by *Bmp2* KD (Figure 5A).

Cardiomyocyte differentiation assays using *lncRNA-Smad7* KIpA, Bmp2 KD/*lncRNA-Smad7* KIpA, *Bmp2* KD or WT mESCs revealed that CP proliferation rates were inhibited under *Bmp2* depletion in both WT and *lncRNA-Smad7* KIpA cells, as indicated by immunofluorescence staining for pH3 on CM D3.3 (Figure 5B, C).

Subsequent quantification of total protein accumulation and immunofluorescence staining for cTnT indicated that *Bmp2* KD could rescue defects in CM maturation in *lncRNA-Smad7* KIpA cells (Figure 5D–F). This result was supported by restoration of increased cardiac-specific marker expression in *lncRNA-Smad7* KIpA/*Bmp2* KD cells (Figure 5G). Collectively, these findings indicated that *Bmp2* KD rescues impaired cardiomyocyte differentiation in *lncRNA-Smad*7-depleted cells. Thus, *Bmp2* is directly repressed by *lncRNA-Smad7* transcriptionally.

Previous studies have reported that *Hand1* is regulated by BMP signaling (23,50). We found that *Hand1* expression was significantly down-regulated in *Bmp2* KD EBs on CM D3.3 (Supplementary Figure S5A) and up-regulated by BMP2 treatment (Supplementary Figure S5B), indicating that *Hand1* might be regulated by a *lncRNA-Smad7*-*Bmp2* axis. We thus established *Hand1* KD cell lines in both *lncRNA-Smad7* KIpA and WT cells (Supplementary Figure S5C). *Hand1* KD led to the enhanced CM maturation, indicated by cTnT protein levels at CM D10 (Supplementary Figure S5D). In addition, cardiac-specific marker gene expression was rescued in *lncRNA-Smad7* KIpA/*Hand1* KD cells (Supplementary Figure S5E). These results together implied that a *lncRNA-Smad7*-*Bmp2* axis may regulate cardiomyocyte differentiation via *Hand1*. However, we observed no association between *lncRNA-Smad7* and the *Hand1* genomic locus using ChIRP-seq analysis, suggesting that *Hand1* was not directly regulated by *lncRNA-Smad7* (Supplementary Figure S5F).

Taken together, *lncRNA-Smad7* directly represses *Bmp2* transcription, thereby prevents expression of *Hand1* which encodes the main factor regulating cardiomyocyte differentiation.

### *LncRNA-Smad7* interacts with hnRNPK and potentially regulates H3K27me3 modification at the *Bmp2* promoter locus

**Figure 6.**
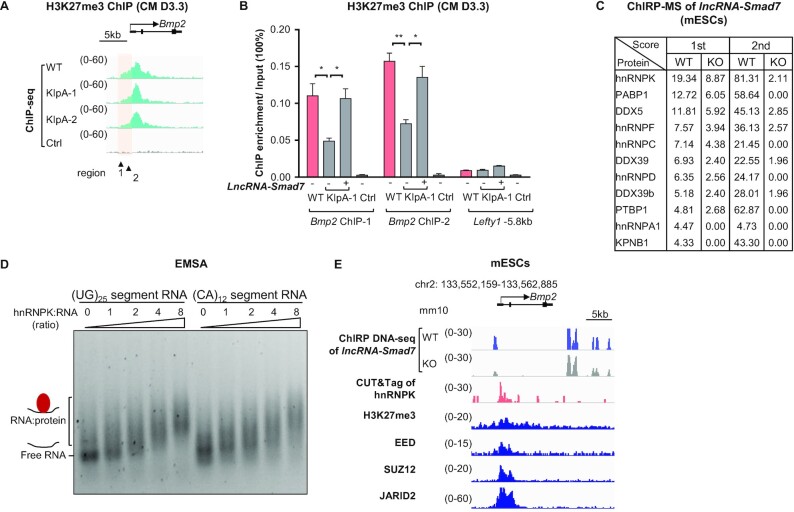
*LncRNA-Smad7* KD leads to decreased H3K27me3 modification at the *Bmp2* promoter region and *lncRNA-Smad7* interacts with hnRNPK. (**A**) IGV tracks showed H3K27me3 ChIP-seq of WT and *lncRNA-Smad7* KIpA progenitor cells on CM D3.3 (mm10). (**B**) ChIP-qPCR analysis of H3K27me3 signals at the promoter regions of *Bmp2* on CM D3.3. The IgG was set for the control group, and –5.8 kb region of *Lefty1* was used as a negative control for H3K27me3 enrichment. (**C**) Combination of mass spectrometry (MS) and ChIRP indicated the interacted proteins of *lncRNA-Smad7* in mESCs with two independent experiments (related to Supplementary Table S6). The KO cells are set as the negative control. (**D**) EMSA showed the association of (UG)_25_ containing and (CA)_12_ containing segments of *lncRNA-Smad7* with hnRNPK *in vitro*. 1.6 pmol RNA were used as a fixed amount in this experiment. (**E**) IGV tracks showed ChIRP-DNA seq of *lncRNA-Smad7*, CUT & Tag of hnRNPK, and ChIP-seq of H3K27me3 and PRC2 complex in mESCs at Bmp2 loci (GSE183465, GSE103258, GSE127117) (mm10). (B) All data are representative of three independent experiments. Data are presented as mean ± S.D., *n* = 3, *P< 0.05, **P < 0.01, ***P < 0.001 (two-tailed Student's *t* test). See also Supplementary Figure S6.

In mESCs, developmental genes are silenced which often carry bivalent mark H3K4me3 and H3K27me3, such as those in the *Bmp2* promoter (63), although they remain poised for activation during differentiation (Supplementary Figure S6A). H3K27me3 modifications, catalyzed by polycomb repressive complex 2 (PRC2), are enriched at the transcriptionally repressed loci of several developmental genes (64), but are removed during early mammalian development (65). Given that *Bmp2* expression was repressed by *lncRNA-Smad7*, we next investigated whether H3K27me3 modifications in the *Bmp2* promoter were maintained through *lncRNA-Smad7*. Consistent with this possibility, ChIP-seq analysis showed that the H3K27me3 signal in the bivalent chromatin domain of *Bmp2* was decreased in *lncRNA-Smad7* KIpA cells compared to that in WT cells on CM D3.3 (Figure 6A). Moreover, the attenuated H3K27me3 signal at the *Bmp2* promoter region was significantly increased by overexpression of *lncRNA-Smad7* in *lncRNA-Smad7* KD cells (Figure 6B). Interestingly, the *lncRNA-Smad7* associates at *Bmp2* promoter (Figure 3H), suggesting that *lncRNA-Smad7* may facilitate H3K27me3 modification at *Bmp2* promoter.

To identify any additional binding partners of *lncRNA-Smad7* that may participate in transcriptional regulation by *lncRNA-Smad7*, we conducted another ChIRP assay followed by mass spectrometry analysis (LC–MS/MS). This experiment revealed that the candidate *lncRNA-Smad7* interacting proteins were RNA-binding proteins (Figure 6C and Supplementary Table S6), among which hnRNPK was the top ranked potential interaction partner based on the score of LC–MS/MS. In addition, RNA immunoprecipitation (RIP) assays published by another group indicated that hnRNPK could indeed interact with *lncRNA-Smad7* in mESCs (Supplementary Figure S6B) (66).

HnRNPK has been described as the principal binding factor responsible for bridging PRC1 with *Xist* and is necessary for PRC1 and PRC2 recruitment (67,68). Moreover, hnRNPK is also required to mediate H3K27me3 modifications across targeted chromatin regions (66). To further examine the direct interaction between hnRNPK and *lncRNA-Smad7*, we incubated purified hnRNPK (Supplementary Figure S6C) with in vitro transcribed segments of *lncRNA-Smad7* containing (CA)_12_ or (UG)_25_ repeats (Supplementary Figure S4D) for electrophoretic mobility shift assays (EMSA). The results showed that *lncRNA-Smad7* segments containing either repeat could directly bind to hnRNPK in a dose-dependent manner (Figure 6D and Supplementary Figure S6D), suggesting that *lncRNA-Smad7* interacts with hnRNPK in vitro. In addition, hnRNPK CUT & Tag assays indicated that hnRNPK associates with the *Bmp2* promoter region (Figure 6E). Interestingly, analysis of published datasets showed that PRC2 and PRC1 complex also associate with promoter region of *Bmp2* that is enriched with *lncRNA-Smad7* and hnRNPK (Figure 6E and Supplementary Figure S6E) (69,70). Thus, hnRNPK may facilitate repression of *Bmp2* by *lncRNA-Smad7* through association with the *Bmp2* promoter, most likely via maintenance of H3K27me3 marks.

### *LncRNA-Smad7* regulates cell fate determination by repressing *Bmp2* in C2C12 cells

**Figure 7.**
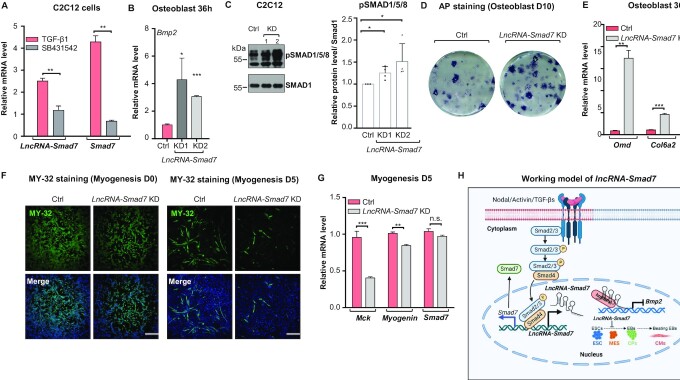
*LncRNA-Smad7* regulates cell fate determination of C2C12 cells. (**A**) qPCR analysis of *lncRNA-Smad7* and *Smad7* expressions in C2C12 cells treated with TGF-β1 (10 ng/ml) or SB431542 (25 μM). (**B**) qPCR analysis of *Bmp2* expression in *lncRNA-Smad7* KD (shRNA) and control C2C12 cells at osteoblast differentiation 36 h. Data are presented as mean ± S.D., *n* = 8, *P < 0.05, **P < 0.01 (two-tailed Student's *t* test). (**C**) Western blot showed the phospho-Smad1/5/8 levels in the two *lncRNA-Smad7* KD cell lines (1 and 2) and control C2C12 cells. Smad1 was used as the loading control; quantification of the phospho-Smad1/5/8 intensities showed in Figure 7C. Scatter plots represent the four biologically independent experiments. Data are presented as mean ± S.D., *P < 0.05 (two-tailed Student's *t* test). (**D**) AP staining of *lncRNA-Smad7* KD and control C2C12 cells on osteoblast differentiation day 10. The data are representative of four independent experiments. (**E**) qPCR analysis of *Smad7* and marker genes (*Omd*, *Col6a2*) in the indicated C2C12 cells on osteoblast differentiation 36 h. (**F**) Immunofluorescence staining showed the MY-32 levels (green) of *lncRNA-Smad7* KD and control C2C12 cells on myogenesis day 0 and day 5. Nuclei were stained with DAPI (blue); scale bar, 100 μm. (**G**) qPCR analysis showed the expressions of *Smad7* and marker genes (*Mck, Myogenin*) in the indicated C2C12 cells on myogenesis differentiation day 5. (**H**) Working model of *lncRNA-Smad7* in cross-talk regulation of TGF-β and BMP signalings. Figure was created in BioRender.com. (A, E, G) All data are representative of three independent experiments. Data are presented as mean ± S.D., **P < 0.01, ***P < 0.001 (two-tailed Student's *t* test). See also Supplementary Figure S7.

The above data led us to hypothesize that this cross-talk mediated by *lncRNA-Smad7* may represent a general regulatory mechanism that is conserved among cells which recognize both TGF-β/Nodal and BMP ligands. C2C12 cells are progenitor mesenchymal cells with the potential to differentiate into osteoblasts or myoblasts. In these cells, BMP signaling is known to reverse cell fate determination from myocytes to osteocytes, while TGF-β signaling can negatively regulate osteoblast formation (63). Thus, using C2C12 cells, we first verified that *lncRNA-Smad7* is responsive to TGF-β signaling (Figure 7A), then we conducted differentiation assays in which C2C12 cells were separately induced to differentiate into either myoblasts or osteoblasts (64,65). QPCR-based analysis indicated that *lncRNA-Smad7* expression levels were significantly elevated during myogenesis compared to that in uninduced C2C12 cells, whereas its expression initially increased then substantially decreased during osteocyte differentiation (Supplementary Figures S7A, B).

In addition to the above trends, relative expression analysis further indicated that *Bmp2* was significantly increased in C2C12 cells with *lncRNA-Smad7* KD (using the same shRNAs as in mESCs) (Figure 7B). Consistent with this finding, phospho-SMAD1/5/8 levels were also substantially increased in *lncRNA-Smad7* KD C2C12 cells (Figure 7C). Moreover, alkaline phosphatase (AP) activity, an osteoblast-specific differentiation marker, was significantly up-regulated in *lncRNA-Smad7* KD cells (Figure 7D). In line with the known functions of BMP signaling in these differentiation processes, the osteoblast differentiation markers *Omd* and *Col6a2* were significantly increased (Figure 7E), while expression levels of the myogenesis differentiation markers, *Mck* and *Myogenin*, were dramatically lower under *lncRNA-Smad7* KD in C2C12 cells (Figure 7F). Additionally, immunofluorescence staining with antibody targeting fast skeletal myosin (MY32) indicated that less myotubes were fused in *lncRNA-Smad7* KD cells compared with WT control cells (Figure 7G and Supplementary Figure S7C). These cumulative results thus expanded the functional relevance of *lncRNA-Smad7* and cross-talk between TGF-β/Nodal and BMP signaling in the cell fate determination of multi-potent progenitor cells.

Collectively, these findings indicated that *lncRNA-Smad7*, regulated by Nodal/TGF-β, transcriptionally represses *Bmp2* to control cell fate determination during early developmental stages. This involvement of *lncRNA-Sma*d7 in the cross-talk between the Nodal/TGF-β and BMP signaling pathways thus defines a layer of regulation beyond that of the known coding genes in these pathways (Figure 7H).

## DISCUSSION

TGF-β (Nodal/Activin/TGF-β) and BMP ligands are present in niches of cells during early development (23,71). Here, we identified the Nodal activated *lncRNA-Smad7*, which associates at the *Bmp2* promoter and represses *Bmp2* expression in mESCs. Hence, Nodal induced *lncRNA-Smad7* mediates cross-talk between Nodal and BMP signaling in mESCs. Moreover, we demonstrated that *lncRNA-Smad7* regulates cardiomyocyte differentiation by repressing *Bmp2*. Interestingly, this cross-talk also applies to the cell fate determination of C2C12 cells. In sum, our study illustrates a *lncRNA-Smad7* mediated cross-talk mechanism between Nodal/TGF-β and BMP signaling in pluripotent and multipotent cells (Figure 7E).

TGF-β family members play essential roles in early development (2,4,72). It is well-established that TGF-β and BMP signaling are antagonistic to each other at different levels (5) and such cross-talk has been reported in various biological processes (73–75). Multiple studies have shown that TGF-β and BMP signaling restricts each other by competing for the limited cellular supply of SMAD4 or R-SMADs (76–79), and TGF-β signaling can suppress BMP signaling by promoting genes expression of BMP signaling antagonists (5,7,8,80). Our data support a model wherein these two pathways are converged via *lncRNA-Smad7* (Figure 7E). This type of the cross-talk represents general phenomena in early development maintaining the balances between different signaling pathways to determine cell fate.

Chromatin associated lncRNAs have been reported to regulate developmental and cellular functions by bridging transcriptional regulators and chromatin (40,81,82). *LncRNA-Smad7* associating with *Bmp2* promoter region and up-regulation of *Bmp2* expression in *lncRNA-Smad7* KD cells suggested that *lncRNA-Smad7* transcriptionally represses *Bmp2* expression. Motif analysis of ChIRP-seq showed that *lncRNA-Smad7* tends to bind at CA- or TG-rich regions of the genome. Interestingly, the *Bmp2* promoter region has (CA:TG)_17_ repeats motif which is overlapped with *lncRNA-Smad7* binding site and the *lncRNA-Smad7* transcript contains both a (CA)_12_- and a (UG)_25_-repeat regions. The in vitro binding assays further confirmed that the (CA)_12_ containing RNA segment of *lncRNA-Smad7* associates with ss (TG)_17_-repeats at the *Bmp2* promoter. Studies showed that RNA could associate with DNA to form the R-loop (58,59) or triplex (60–62). Thus, it is worth investigating how exactly *lncRNA-Smad7* associates with this chromatin region through structural biology to elucidate the complex of *lncRNA-Smad7* with the *Bmp2* promoter.

Polycomb complex is required for maintaining pluripotency in embryonic stem cells (83,84). Many lncRNAs have been shown to function as transcriptional repressive factors (66,85,86) by recruiting the polycomb complex to the target genes (36). Here, we have demonstrated that *lncRNA-Smad7* is essential to maintain the H3K27me3 modification in the *Bmp2* promoter region. And hnRNPK was validated to directly bind with *lncRNA-Smad7* in vitro. Since hnRNPK was reported to function as a bridge between lncRNA and polycomb complex by associating with both factors (66,67), it is likely that *lncRNA-Smad7* represses *Bmp2* expression by recruiting hnRNPK at the *Bmp2* promoter region, consequently bringing transcriptional repressive factors (*e.g*. polycomb complex) to the locus. In the future, elucidating the assembling of *lncRNA-Smad7*, the hnRNPK, and polycomb complex at *Bmp2* promoter region is worth the investigation.

Mouse *lncRNA-Smad7* is not conserved among species based on the primary sequences. However, RNA molecules are folded into complex of three-dimensional structures which is more conserved than its primary sequences, and RNA structures often play essential roles in RNAs’ function (39,40). It is possible that there are RNAs in other species with similar function as mouse *lncRNA-Smad7* which might share the conserved structure but not primary sequences. In addition, our data showed that (CA)_12_-repeats in *lncRNA-Smad7* is essential for the binding of *lncRNA-Smad7* to the *Bmp2* promoter region. There are some CA-repeats containing lncRNAs in human, and we speculate that there may be CA-repeats containing lncRNAs which function similarly to mouse *lncRNA-Smad7* in Homo sapiens (Supplementary Table S5). Recent studies showed more techniques have been developed to probe the RNA structure and its RNA binding proteins (87–89), especially for those highly abundant RNA. If these techniques allow to detect the structure of the low abundant RNA like *lncRNA-Smad7*, it is worth taking advantage of these techniques to reveal the RNA structure in the future.

Given that *lncRNA-Smad7* and *Smad7* share the same promoter (89) and there are potential regulatory elements of *Smad7* embedded in the *lncRNA-Smad7* locus, it is not feasible to generate *lncRNA-Smad7* KO mice by truncating the whole locus of *lncRNA-Smad7* since the truncation will affect *Smad7* expression. Once the functional domain of *lncRNA-Smad7* is identified, it would be a plausible way for us to generate KO mice by deleting the functional domain of *lncRNA-Smad7*, which will elucidate the function of *lncRNA-Smad7* in early development.

## DATA AVAILABILITY

Raw data have been deposited in the GEO database with the series accession GSE178136. The mass spectrometry proteomics data have been deposited to the ProteomeXchange Consortium via the PRIDE partner repository with the dataset identifier PXD036394.

## Supplementary Material

gkac780_Supplemental_Files
