# Correction to ‘*LncRNA-Smad7* mediates cross-talk between nodal/TGF-β and BMP signaling to regulate cell fate determination of pluripotent and multipotent cells’

**DOI:** 10.1093/nar/gkad173

**Published:** 2023-03-07

*Nucleic Acids Research*, Volume 50, Issue 18, 14 October 2022, Pages 10526–10543, https://doi.org/10.1093/nar/gkac780

In the originally published version of this manuscript, the following reference was omitted:

Maldotti, M., Lauria, A., Anselmi, F., Molineris, I., Tamburrini, A., Meng, G., Polignano, I.L., Scrivano, M.G., Campestre, F., Simon, L.M. *et al.* (2022) The acetyltransferase p300 is recruited in trans to multiple enhancer sites by lncSmad7. *Nucleic Acids Res*., **50**, 2587–2602.

This reference has now been added to the online version of the paper as reference 89.

