# Supplementary material for: LncRNA-Smad7 mediates cross-talk between Nodal/TGF-β and BMP signaling to regulate cell fate determination of pluripotent and multipotent cells
Source: Nucleic Acids Res. 2022 Sep 22;50(18):10526–43. doi: 10.1093/nar/gkac780 (PMC9561265; doi:10.1093/nar/gkac780)
Supplement: gkac780_Supplemental_Files [file gkac780_supplemental_files.zip › Supplementary figures.pdf]

Supplementary information:

LncRNA-Smad7 mediates cross-talk between Nodal/TGF-β and BMP signaling to regulate cell fate determination of pluripotent and multipotent cells

Xiaohui Kong, Kun Yan, Pujuan Deng, Haipeng Fu, Hongyao Sun, Wenze Huang, Shuangying Jiang, Junbiao Dai, Qiangfeng Cliff Zhang, Jun-jie Gogo Liu, Qiaoran Xi

1. Supplementary figures (S1-S7)

2. Supplemental figure legends

3. Supplementary methods and materials

4. Supplementary tables (S1-S6)

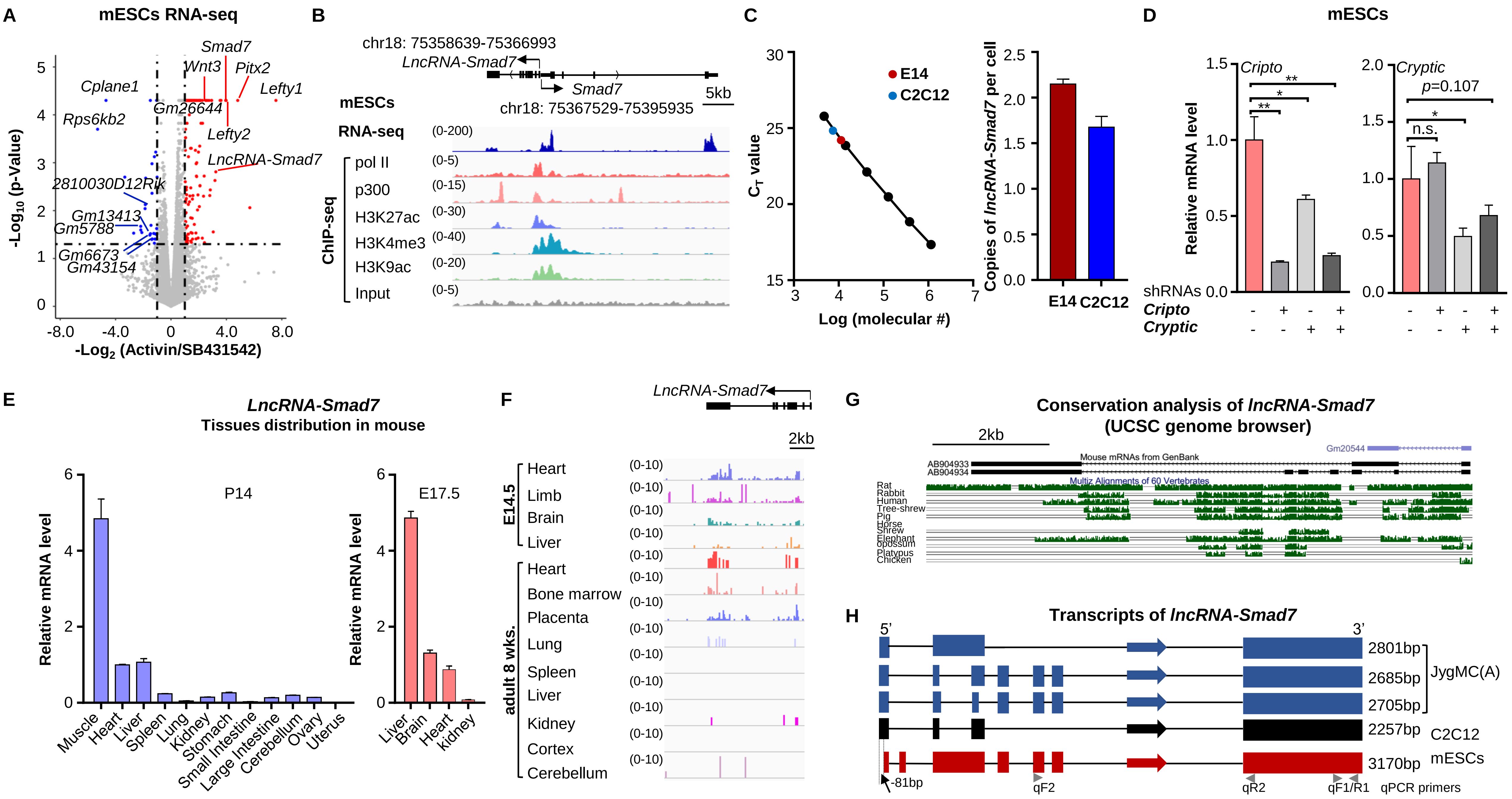

A
mESCs RNA-seq
Smad7
Wnt3
Pitx2
Lefty1
Cplane1
Gm26644
Rps6kb2
Lefty2
LncRNA-Smad7
2810030D12Rik
Gm13413
Gm5788
Gm6673
Gm43154
-Log10 (p-Value)
-Log2 (Activin/SB431542)
B
chr18: 75358639-75366993
LncRNA-Smad7
Smad7
5kb
chr18: 75367529-75395935
mESCs
RNA-seq
ChIP-seq
pol II
p300
H3K27ac
H3K4me3
H3K9ac
Input
C
E14
C2C12
CT value
Log (molecular #)
Copies of lncRNA-Smad7 per cell
D
mESCs
Cripto
Cryptic
p=0.107
n.s.
Relative mRNA level
shRNAs
E
LncRNA-Smad7
Tissues distribution in mouse
P14
E17.5
Relative mRNA level
Muscle
Heart
Liver
Spleen
Lung
Kidney
Stomach
Small Intestine
Large Intestine
Cerebellum
Ovary
Uterus
Brain
kidney
F
LncRNA-Smad7
2kb
E14.5
Heart
Limb
Brain
Liver
adult 8 wks.
Bone marrow
Placenta
Lung
Spleen
Kidney
Cortex
Cerebellum
G
Conservation analysis of lncRNA-Smad7
(UCSC genome browser)
2kb
Gm20544
Mouse mRNAs from GenBank
AB904933
AB904934
Multiz Alignments of 60 Vertebrates
Rat
Rabbit
Human
Tree-shrew
Pig
Horse
Shrew
Elephant
opossum
Platypus
Chicken
H
Transcripts of lncRNA-Smad7
5'
3'
2801bp
2685bp
2705bp
JygMC(A)
2257bp
C2C12
3170bp
mESCs
-81bp
qF2
qR2
qF1/R1
qPCR primers

Supplementary Figure S1. Characterization of *lncRNA-Smad7*. Related to Figure 1.

(A) Volcano plot of the DEGs (2-fold cutoff) in Activin A treated mESCs compared with SB431542 treated mESCs. The data are representative of two independent experiments (GSE115169).

(B) IGV tracks of the ChIP-seq of RNA polymerase II, p300 (GSE36027) and the active histone modification H3K27ac, H3K4me3, H3K9ac (GSE31039) at *lncRNA-Smad7* and *Smad7* loci in mESCs (mm9).

(C) The copy number of *lncRNA-Smad7* per cell in E14 and C2C12 cells. Left, the linear relationship between the log *lncRNA-Smad7* copy number and its C_T value by qRT-PCR. Black dots represent known copies of *lncRNA-Smad7* from a plasmid DNA containing *lncRNA-Smad7* sequences; and the red and blue dots represent *lncRNA-Smad7* copies in indicated cells. Right, the average copies of *lncRNA-Smad7* per cell.

(D) qPCR analysis of *Cripto* and *Cryptic* expressions in *Cripto* KD, *Cryptic* KD, and *Cripto*/*Cryptic* double KD mESCs.

(E) Histograms showing the relative expression level of *lncRNA-Smad7* in the indicated tissues from postnatal day 14 (P14) mice and day 17.5 embryos (E17.5).

(F) IGV tracks of lncRNA-Smad7 expression in various tissues in embryo E14.5 and postnatal week 8 (adult 8 wks.) mice (GSE29278) (mm9).

(G) Cross-species genome conservation analysis of *lncRNA-Smad7* among mouse, human and other species. *Gm20544*, *AB904933*, and *AB904934* are all previously reported as different isoforms of *lncRNA-Smad7.*

(H) Summary of the different transcripts of *lncRNA-Smad7* in breast cancer cells (blue), C2C12 cell (black), and mESCs (red) (1,2).

Data are presented as mean ± S.D., n = 3, asterisks indicate a difference from control (two-tailed Student's *t* test). * $p < 0.05$; n.s., not significant.

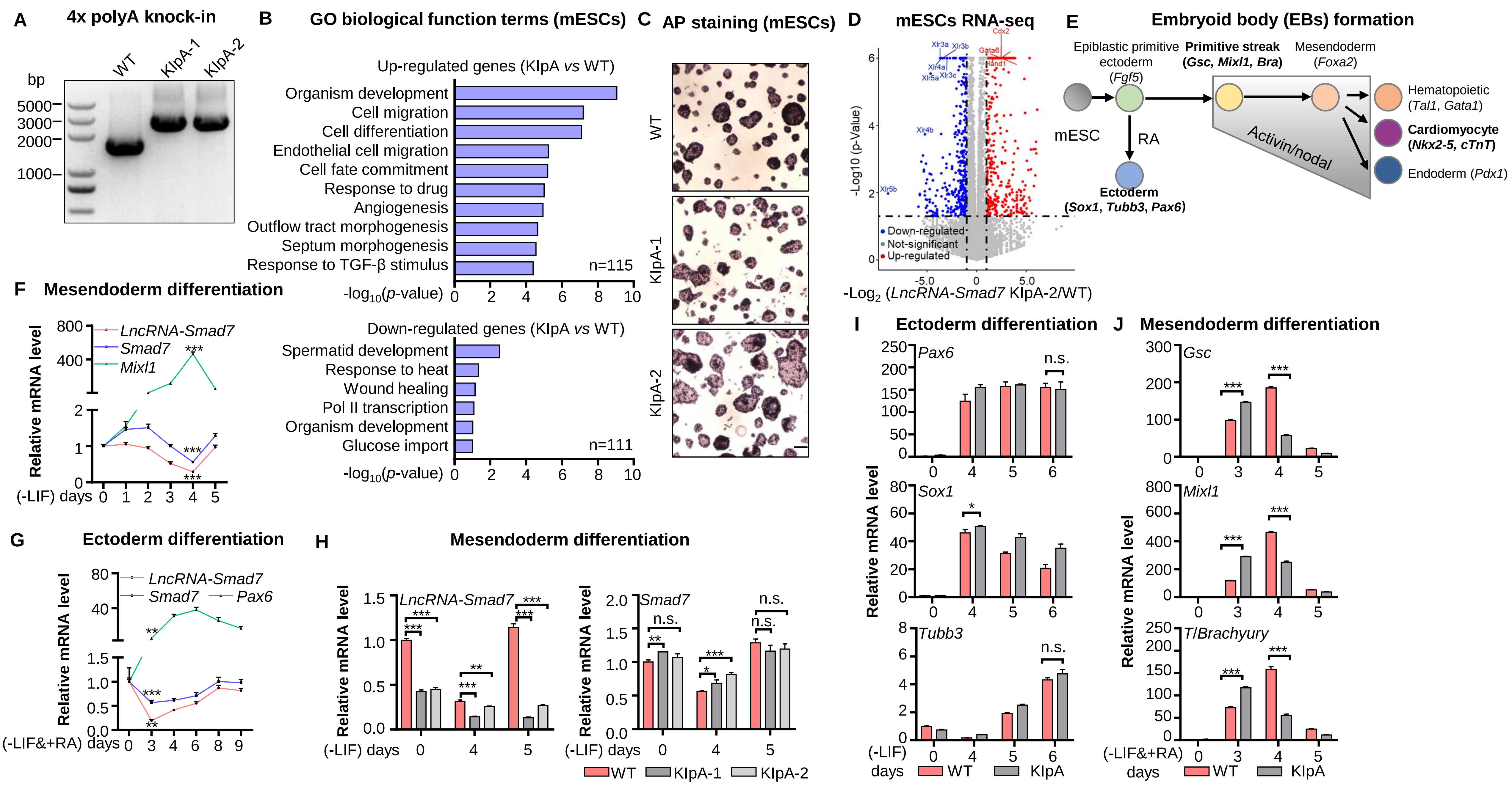
A
4x polyA knock-in
WT
KlpA-1
KlpA-2
bp
5000
3000
2000
1000
B
GO biological function terms (mESCs)
Up-regulated genes (KlpA vs WT)
Organism development
Cell migration
Cell differentiation
Endothelial cell migration
Cell fate commitment
Response to drug
Angiogenesis
Outflow tract morphogenesis
Septum morphogenesis
Response to TGF-β stimulus
n=115
-log10(p-value)
Down-regulated genes (KlpA vs WT)
Spermatid development
Response to heat
Wound healing
Pol II transcription
Organism development
Glucose import
n=111
-log10(p-value)
C
AP staining (mESCs)
WT
KlpA-1
KlpA-2
D
mESCs RNA-seq
-Log10 (p-Value)
Down-regulated
Not-significant
Up-regulated
-Log2 (LncRNA-Smad7 KlpA-2/WT)
E
Embryoid body (EBs) formation
Epiblastic primitive ectoderm (Fgf5)
Primitive streak (Gsc, Mixl1, Bra)
Mesendoderm (Foxa2)
mESC
RA
Ectoderm (Sox1, Tubb3, Pax6)
Activin/nodal
Hematopoietic (Tal1, Gata1)
Cardiomyocyte (Nkx2-5, cTnT)
Endoderm (Pdx1)
F
Mesendoderm differentiation
Relative mRNA level
LncRNA-Smad7
Smad7
Mixl1
(-LIF) days
G
Ectoderm differentiation
Relative mRNA level
LncRNA-Smad7
Smad7
Pax6
(-LIF&+RA) days
H
Mesendoderm differentiation
Relative mRNA level
LncRNA-Smad7
Smad7
(-LIF) days
WT
KlpA-1
KlpA-2
I
Ectoderm differentiation
Relative mRNA level
Pax6
Sox1
Tubb3
(-LIF) days
WT
KlpA
J
Mesendoderm differentiation
Relative mRNA level
Gsc
Mixl1
T/Brachyury
(-LIF&+RA) days
WT
KlpA

Supplementary Figure S2. *LncRNA-Smad7* KD impairs mesendoderm differentiation of mESCs. Related to Figure 2.

(A) PCR-based genotyping to validate the *lncRNA-Smad7* KIpA and wide type (WT) mESCs. The amplicon length of WT and KIpA were 1850bp and 3000bp.

(B) GO terms analysis of significant DEGs in *lncRNA-Smad7* KIpA and WT mESCs (up-regulated genes, 2-fold cutoff, n = 115; down-regulated genes, 2-fold cutoff, n = 111). The data are representative of two independent experiments.

(C) Alkaline phosphatase staining (AP staining) of *lncRNA-Smad7* KIpA and WT mESCs. The data are representative of three independent experiments; scale bar, 50 μm.

(D) Volcano plot of the DEGs (2-fold cutoff) in *lncRNA-Smad7* KIpA-2 cells compared with WT mESCs. The data are representative of two independent experiments.

(E) Schematic summary of mESCs differentiation by EBs formation in vitro (3,4).

(F) qPCR analysis of the indicated transcripts during mesendoderm differentiation. Asterisks indicate a difference of gene expressions on day 4 relative to day 0.

(G) qPCR analysis of the indicated transcripts during ectoderm differentiation. Asterisks indicate a difference of gene expressions on day 3 relative to day 0.

(H) qPCR analysis of *lncRNA-Smad7* and *Smad7* expressions during mesendoderm differentiation in *lncRNA-Smad7* KIpA and WT cells.

(I) qPCR analysis of the indicated genes expression (i.e., *Pax6*, *Sox1*, and *Tubb3*) in *lncRNA-Smad7* KIpA and WT cells on the indicated days.

(J) qPCR analysis of the indicated genes expression (i.e., *Gsc*, *Mixl1*, and *T/Brachyury*) in *lncRNA-Smad7* KIpA and WT cells on the indicated days.

Figure S2F-J: all data are representative of three independent experiments. Data are presented as mean ± S.D., n = 3, **p* < 0.05, ***p* < 0.01, ****p* < 0.001, n.s., not significant (two-tailed Student's *t* test).

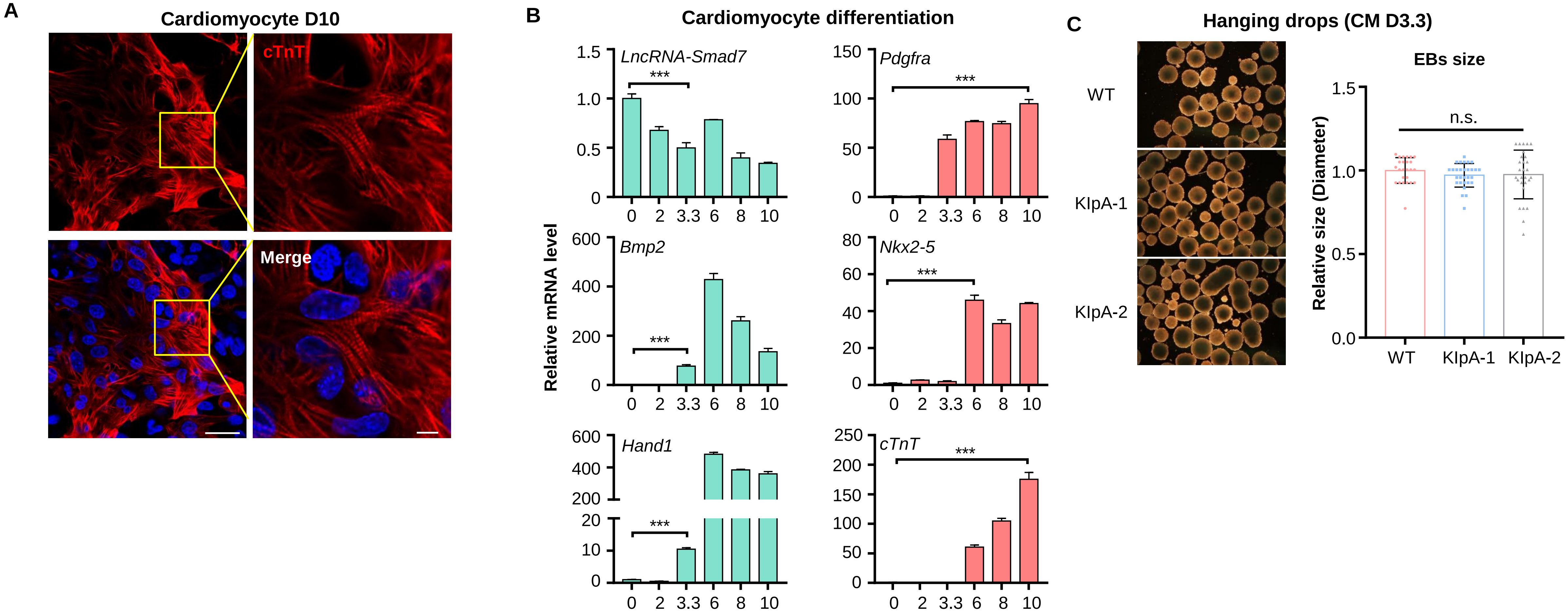
A
Cardiomyocyte D10
cTnT
Merge
B
Cardiomyocyte differentiation
Relative mRNA level
LncRNA-Smad7
Pdgfra
Bmp2
Nkx2-5
Hand1
cTnT
***
0 2 3.3 6 8 10
C
Hanging drops (CM D3.3)
WT
KIpA-1
KIpA-2
EBs size
Relative size (Diameter)
n.s.

Supplementary Figure S3. In vitro cardiomyocyte differentiation. Related to Figure 2.

(A) Immunofluorescence staining of cTnT (red) of WT CMs on D10. Nuclei were stained with DAPI (blue); scale bar, 25 μm and 10 μm.

(B) qPCR analysis of the indicated transcripts expression in cardiomyocyte differentiation of mESCs. Asterisks indicate a difference of genes expression on the indicated days relative to day 0.

(C) Images showing EBs size at CM D3.3 for indicated cells (left panels); the size quantification of EBs was shown in the right panels; scale bar, 50μm. The data are representative of three independent experiments, data are presented as mean ± S.D., n ≥ 25 (two-tailed Student's *t* test). n.s., not significant.

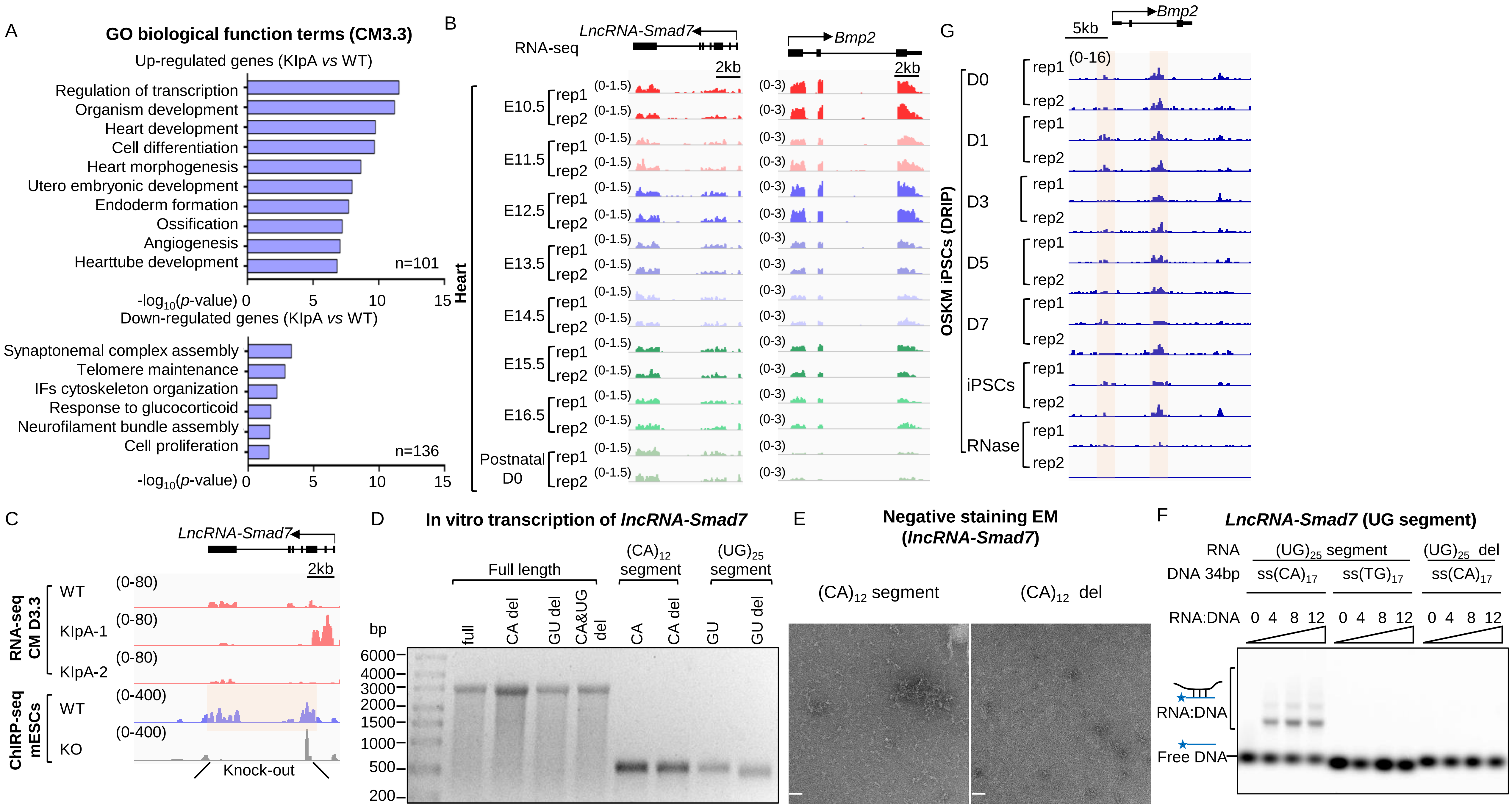
A
GO biological function terms (CM3.3)
Up-regulated genes (KIpA vs WT)
Regulation of transcription
Organism development
Heart development
Cell differentiation
Heart morphogenesis
Utero embryonic development
Endoderm formation
Ossification
Angiogenesis
Hearttube development
n=101
-log10(p-value) 0 5 10 15
Down-regulated genes (KIpA vs WT)
Synaptonemal complex assembly
Telomere maintenance
IFs cytoskeleton organization
Response to glucocorticoid
Neurofilament bundle assembly
Cell proliferation
n=136
-log10(p-value) 0 5 10 15
B
RNA-seq
LncRNA-Smad7
Bmp2
2kb
Heart
E10.5 rep1 rep2
E11.5 rep1 rep2
E12.5 rep1 rep2
E13.5 rep1 rep2
E14.5 rep1 rep2
E15.5 rep1 rep2
E16.5 rep1 rep2
Postnatal D0 rep1 rep2
(0-1.5)
(0-3)
G
5kb
Bmp2
(0-16)
OSKM iPSCs (DRIP)
D0 rep1 rep2
D1 rep1 rep2
D3 rep1 rep2
D5 rep1 rep2
D7 rep1 rep2
iPSCs rep1 rep2
RNase rep1 rep2
C
LncRNA-Smad7
2kb
RNA-seq CM D3.3
WT (0-80)
KIpA-1 (0-80)
KIpA-2 (0-80)
ChIRP-seq mESCs
WT (0-400)
KO (0-400)
Knock-out
D
In vitro transcription of lncRNA-Smad7
Full length
(CA)12 segment
(UG)25 segment
full
CA del
GU del
CA&UG del
CA
CA del
GU
GU del
bp
6000
4000
3000
2000
1500
1000
500
200
E
Negative staining EM (lncRNA-Smad7)
(CA)12 segment
(CA)12 del
F
LncRNA-Smad7 (UG segment)
RNA
(UG)25 segment
(UG)25 del
DNA 34bp
ss(CA)17
ss(TG)17
ss(CA)17
RNA:DNA 0 4 8 12
RNA:DNA
Free DNA

Supplementary Figure S4. *LncRNA-Smad7* binds at *Bmp2* promoter during cardiomyocyte differentiation. Related to Figure 3 and Figure 4.

(A) GO terms analysis of the significant DEGs (2-fold cutoff) in *lncRNA-Smad7* KIpA cells compared to WT on CM D3.3 (up-regulated genes, n = 101; down-regulated genes, n = 136). The data are representative of two independent experiments.

(B) IGV tracks showed expressions of *lncRNA-Smad7* and *Bmp2* in embryo hearts from E10.5 to E16.5, and the new-born mice (5) (mm10).

(C) IGV tracks showed RNA-seq of *lncRNA-Smad7* KIpA and WT cells on CM D3.3; ChIRP-seq of *lncRNA-Smad7* in WT and *lncRNA-Smad7* KO mESCs at *lncRNA-Smad7* loci (mm10).

(D) In vitro transcribed RNA of the full length and segments of *lncRNA-Smad7* as indicated. bp, base pairs.

(E) Negative staining EM showed the structures of indicated *lncRNA-Smad7* segments in vitro; scale bar, 50nm.

(F) EMSA showed binding affinity of 34bp ss $(TG)_{17}$ or ss $(CA)_{17}$ repeats with the increasing amounts of $(UG)_{25}$ containing segment and $(UG)_{25}$-del segment of *lncRNA-Smad7*.

(G) IGV tracks showed DRIP-seq of S9.6 in mouse iPSCs process induced by OSKM (GSE125644) (mm10).

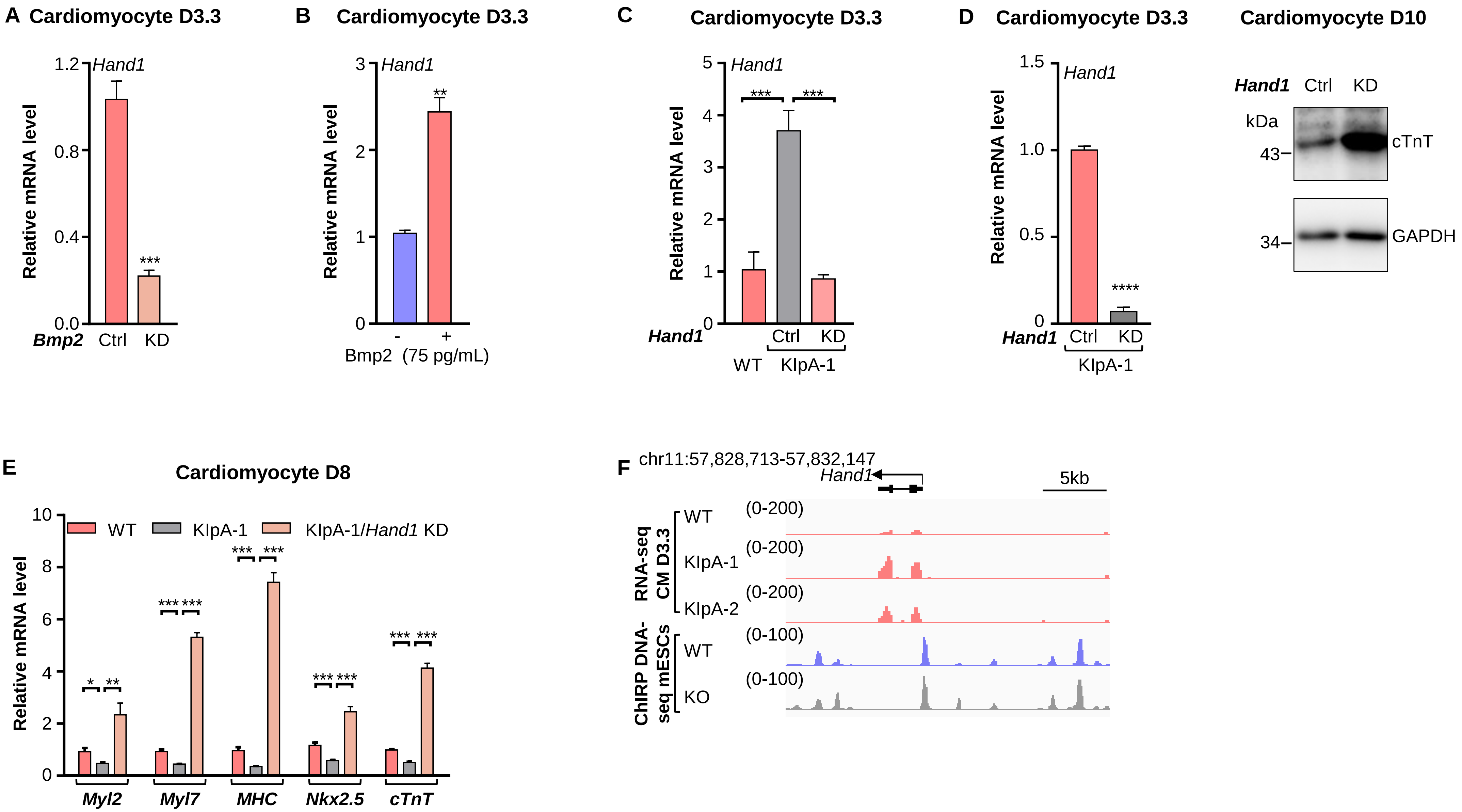
A Cardiomyocyte D3.3
Hand1
Relative mRNA level
1.2
0.8
0.4
0.0
***
Bmp2 Ctrl KD
B Cardiomyocyte D3.3
Hand1
Relative mRNA level
3
2
1
0
**
- +
Bmp2 (75 pg/mL)
C Cardiomyocyte D3.3
Hand1
Relative mRNA level
5
4
3
2
1
0
*** ***
Hand1 Ctrl KD
WT KIpA-1
D Cardiomyocyte D3.3
Hand1
Relative mRNA level
1.5
1.0
0.5
0
****
Hand1 Ctrl KD
KIpA-1
Cardiomyocyte D10
Hand1 Ctrl KD
kDa
43–
cTnT
34–
GAPDH
E Cardiomyocyte D8
Relative mRNA level
10
8
6
4
2
0
WT KIpA-1 KIpA-1/Hand1 KD
* ** *** *** *** *** *** *** *** ***
Myl2 Myl7 MHC Nkx2.5 cTnT
F chr11:57,828,713-57,832,147
Hand1
5kb
RNA-seq CM D3.3
WT (0-200)
KIpA-1 (0-200)
KIpA-2 (0-200)
ChIRP DNA-seq mESCs
WT (0-100)
KO (0-100)

Supplementary Figure S5. *LncRNA-Smad7-Bmp2-Hand1* axis in cardiomyocyte differentiation. Related to Figure 5.

(A) qPCR analysis of *Hand1* expression in *Bmp2* KD cells on CM D3.3.

(B) qPCR analysis of *Hand1* expression with Bmp2 treatment (75 pg/mL) or not in WT cells on CM D3.3.

(C) Knock-down efficiency of the shRNA against *Hand1* on CM D3.3.

(D) (Left) qPCR showed the knock-down efficiency of Hand1 in cardiomyocyte day 3.3. (Right) Western blot showed the protein level of cTnT in *Hand1* KD and control CMs on day 10 (D10). GAPDH was used as the loading control.

(E) qPCR analysis of key cardiac-specific genes in *lncRNA-Smad7* KIpA, *lncRNA-Smad7* KIpA/*Hand1* KD, and control CMs on day 8 (D8).

(F) IGV tracks showed RNA-seq of *lncRNA-Smad7* KIpA and WT cells on CM D3.3; ChIRP-seq of *lncRNA-Smad7* in WT and *lncRNA-Smad7* KO mESCs at *Hand1* loci (mm10).

All data are representative of three independent experiments, data are presented as mean ± S.D., n = 3 (two-tailed Student's t test). *$p < 0.05$; **$p < 0.01$; *** $p < 0.001$.

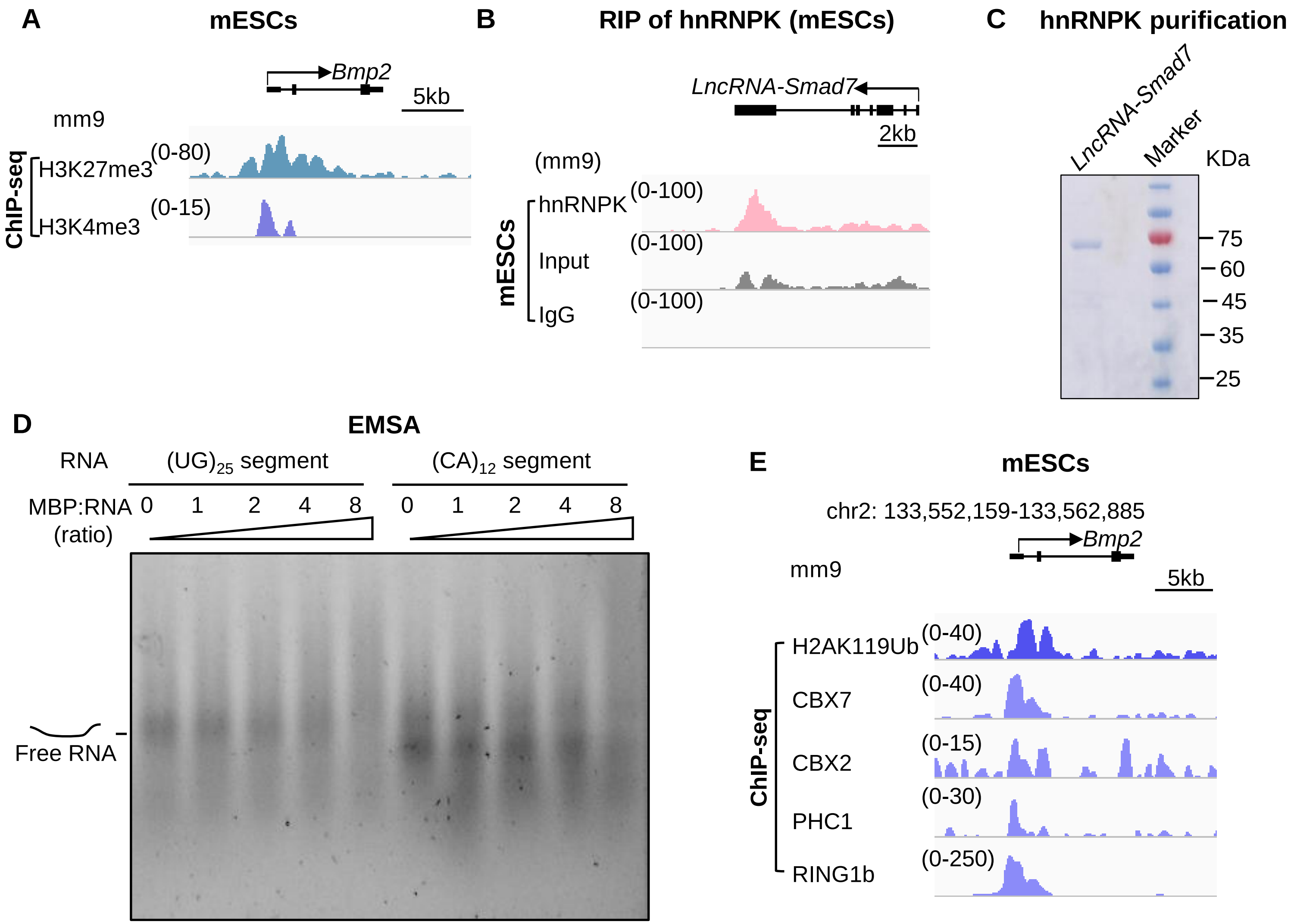
A
mESCs
Bmp2
5kb
mm9
ChIP-seq
H3K27me3
(0-80)
H3K4me3
(0-15)
B
RIP of hnRNPK (mESCs)
LncRNA-Smad7
2kb
(mm9)
mESCs
hnRNPK
(0-100)
Input
(0-100)
IgG
(0-100)
C
hnRNPK purification
LncRNA-Smad7
Marker
KDa
75
60
45
35
25
D
EMSA
RNA
(UG)25 segment
(CA)12 segment
MBP:RNA (ratio)
0 1 2 4 8
0 1 2 4 8
Free RNA
E
mESCs
chr2: 133,552,159-133,562,885
Bmp2
mm9
5kb
ChIP-seq
H2AK119Ub
(0-40)
CBX7
(0-40)
CBX2
(0-15)
PHC1
(0-30)
RING1b
(0-250)

Supplementary Figure S6. *LncRNA-Smad7* interacts with hnRNPK in vitro. Related to Figure 6.

(A) IGV tracks showed ChIP-seq of H3H27me3 and H3K4me3 in mESCs at *Bmp2* locus (GSE89929, GSE31039) (mm9).

(B) IGV tracks showed RIP (RNA immunoprecipition) of hnRNPK (GSE129496) in mESCs (sm33) at *lncRNA-Smad7* loci (mm9) (6).

(C) Coomassie blue staining of purified hnRNPK proteins from HEK293T cells.

(D) EMSA showed the association of $(UG)_{25}$ containing and $(CA)_{12}$ containing segments of *lncRNA-Smad7* with MBP (Maltose binding protein) in vitro. 1.6 pmol RNA were used as a fixed amount in this experiment.

(E) IGV tracks showed ChIP-seq of H2AK119Ub and PRC1 complex in mESCs at *Bmp2* loci (GSE89929) (mm9).

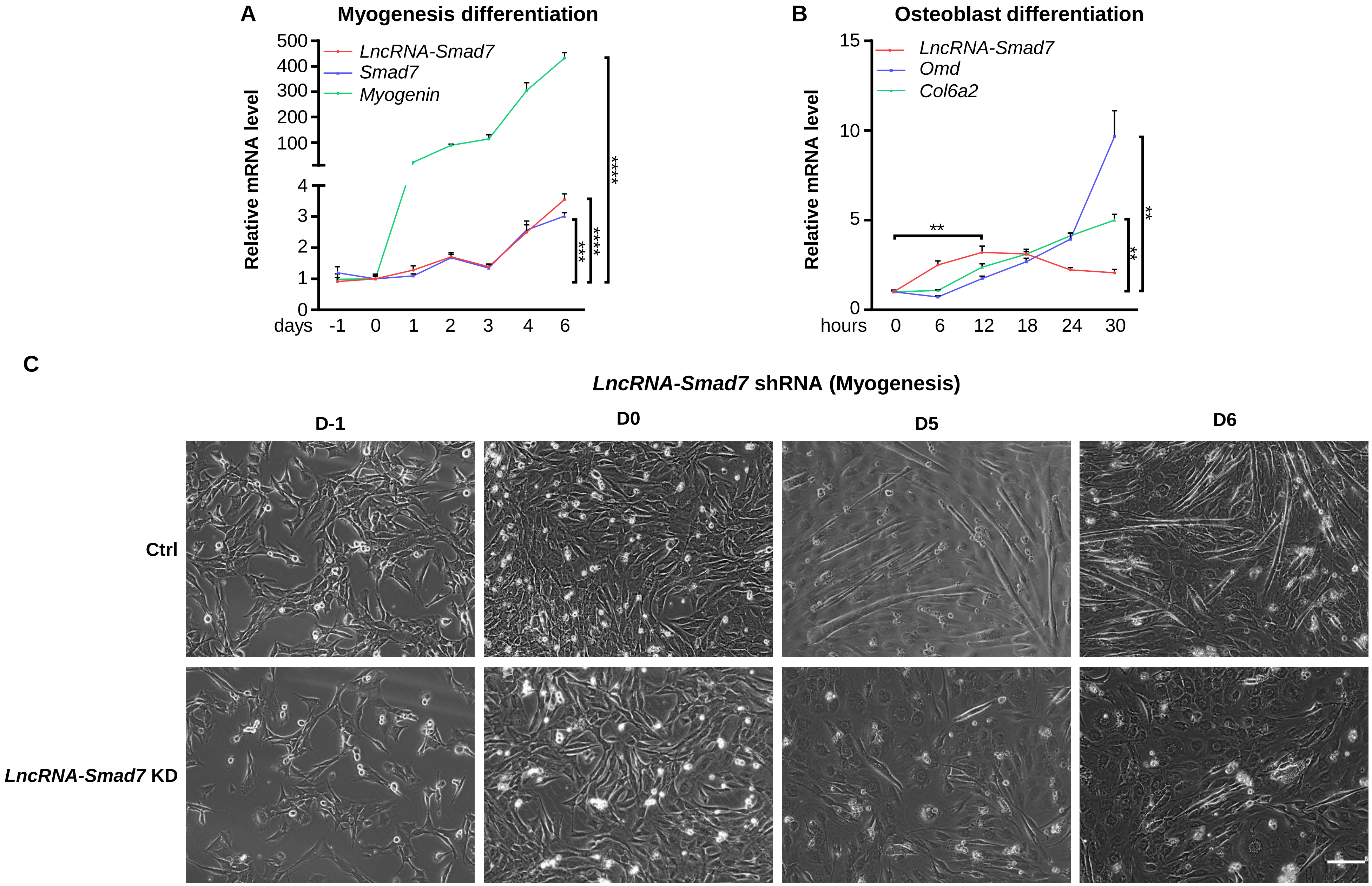
A
Myogenesis differentiation
LncRNA-Smad7
Smad7
Myogenin
Relative mRNA level
500
400
300
200
100
4
3
2
1
0
days -1 0 1 2 3 4 6
***
****
****
B
Osteoblast differentiation
LncRNA-Smad7
Omd
Col6a2
Relative mRNA level
15
10
5
0
hours 0 6 12 18 24 30
**
**
**
C
LncRNA-Smad7 shRNA (Myogenesis)
D-1
D0
D5
D6
Ctrl
LncRNA-Smad7 KD

Supplementary Figure S7. The function of *lncRNA-Smad7* in C2C12 cells. Related to Figure 7.

(A) qPCR analysis of the indicated genes expression during myogenesis differentiation in C2C12 cells. Asterisks indicate a difference of gene expressions on day 6 relative to day 0.

(B) qPCR analysis of the indicated genes during osteoblast differentiation in C2C12 cells. Asterisks indicate a difference of gene expressions on the indicated time relative to 0 hour.

(C) Morphological changes in *lncRNA-Smad7* KD and control C2C12 cells during myogenesis at day -1 (pre differentiation) and then at day 0, day 5, and day 6; scale bar, 200 μm.

Supplementary methods and materials

Generation of shRNA mediated knock-down cells

Small hairpins (shRNAs) were designed by Public TRC Portal (the RNAi Consortium, Broad Institute) or as previously reported. Annealed oligonucleotides of shRNAs were cloned into AgeI and EcoRI digested pLKO.1-puro lentiviral vector driven by U6 promoter. Scramble shRNA was used as the control shRNA. The shRNAs were transfected into HEK293T cells to package lentivirus by lipofectamine 2000 (Invitrogen, Cat# 2270695) followed by infections of mESCs or C2C12 cells. After drug selection, cells were collected for RNA extraction and qPCR analysis. Sequence of shRNAs for *lncRNA-Smad7*, *Bmp2*, and *Hand1* were listed in Supplementary Table S2.

Generation of *lncRNA-Smad7* knock-out and 4x polyA-knock-in (KIpA) cells

The small guide RNAs (sgRNAs) were inserted into BbsI-linearized pSpCas9 (BB)-2A-GFP (PX458) vector (Addgene# 48138) respectively. The sgRNAs were designed using CRISPR design website (http://crispr.mit.edu/). SgRNA for the construction of 4x polyA knock-in mESCs is located at the 47 bp region downstream from 5' end of exon1. The 4x polyA transcription stop cassette containing the SV40 polyA and BGH polyA signal sequence was cloned into the pMD19-T (TaKaRa) donor vector flanked by about 1 kb homologous arms of both upstream and downstream sequence from the sgRNA site respectively (8). We co-transfected the PX458 and donor vector containing homologous arms and 4x polyA sequence at a ratio of 1:4. The sgRNAs of *lncRNA-Smad7* depleted mESCs were designed in the exon1 and exon7 separately. All sgRNAs sequences are listed in Supplementary Table S2.

Cells were transfected using lipofectamine 2000 and GFP-positive cells were FACS sorted as single cells into 96-well plate at 48 hours after transfection. The cells were cultured for 2 weeks followed by PCR-based genotyping. One clone showing deletion of the targeted region in *lncRNA-Smad7* genomic DNA and two clones showing 4x polyA insertion were picked up for further analysis.

Proliferation analysis

EBs of cardiomyocyte on day 3.3 (CM D3.3, 80 hours) were collected and washed with PBS twice, then fixed in 4% paraformaldehyde (PFA) for 30 min at room temperature (RT). The EBs were collected to the 30% sucrose solution for 1-2 days to enable most EBs to sink to the bottom. We embedded the EBs with O.C.T. compound (Sakura, Cat# 4583) to prepare the frozen samples and sectioned them to 7-9 μm with cryostat (Leica, Cat# CM1950). EB sections were permeabilized in 0.5% Triton X-100 in PBS (PBST) for 30 min at RT, then blocked in PBST containing 1% BSA for 1 hour at RT. The sections were incubated in primary antibody anti-pH3 (Abclonal, Cat# AP0840, 1:100 dilution) overnight and AlexaFluor-488-conjugated secondary antibody (huaxingbio, Cat# HX2081) for 1 hour at RT, then counterstained with DAPI (Solarbio, Cat# C0060) for 5 min at RT.

Signals were detected with A1/SIM/STORM-confocal (Nikon, 17018750). Proliferation index was calculated as the ratio of intensity of pH3-positive nuclei to the total cell area. The area was counted on non-consecutive sections per samples by DAPI using NIS (Nikon Imaging Software). Statistical analyses were performed using unpaired two-tailed *t*-test. Data are presented as mean ± S.D. (Standard Deviation), differences were considered statistically significance at $p < 0.05$.

Immunofluorescence of cTnT

EBs were seeded on 35 mm confocal dish (Cellvis, Cat# D35-20-1-N) on day 3.3 and the immunofluorescence staining of CMs were performed on day 10. CMs were fixed with 4% PFA for 10 min, permeabilized with 0.5% Triton X-100 in PBS for 10 min, followed by 1 hour blocking in 1% BSA at RT. CMs were stained with the primary antibody cTnT (Abcam, Cat# ab8295, 1:100 dilution) overnight at 4°C and AlexaFluor-594-conjugated secondary antibody (huaxingbio, Cat# HX2074) for 1 hour at RT. Cells were further processed using DAPI for 5 min at RT. Signals were detected with A1R confocal microscopy (A1/SIM/STORM, Nikon, 17018750; HD25, Nikon).

RT-PCR

The data in this study are presented as mean ± S.D. from three independent experiments at least ($n \geq 3$). *β-actin* (*Actb*) was used as the internal control for qPCR analysis. Significance *, **, *** represents $p < 0.05$, $p < 0.01$, $p < 0.001$, respectively (two-tailed Student's *t* test). Statistical parameters for each experiment, including values of n and statistical significance, can be found in the figure legends.

ChIP-seq and ChIRP-seq analysis

High-throughput sequencing was performed by Novogene on a Hiseq X. The resulting FASTQ files were trimmed using TrimGalore (9) and then aligned to mm10 using Bowtie2 (10). PCR duplicates were removed using PicardTools. We chosed MACS2 to call the peaks and subsequently generated bigwig files from the bam files using the Coverage function in deepTools (11). For visualization purposes, we normalized the data to 1x genome coverage (mm10), ignoring PCR duplicates. Representative track diagrams were generated using the Integrated Genomics Viewer software (12).

Supplementary Table S1. List of qPCR primers.

Gene	Forward (5’-3’)	Reverse (5’-3’)
β-actin	CACAGCTTCTTTGCAGCTCCTT	CGTCATCCATGGCGAACTG
LncRNA-Smad7	CAACCCAATGAGGGGGTCTC	GGCAGTGGACAGACGAATCA
Smad7	ACCCCCATCACCTTAGTCG	GAAAATCCATTGGGTATCTGGA
Myl2	AAAGAGGCTCCAGGTCCAAT	CCTCTCTGCTTGCGTGGTTA
Myl7	CCCATCAACTTCACCGTCTTCCT	AGAGAACTTGTCTGCCTGGGTCA
cTnT	GAGAGAGAAGGCCAAGGAGC	CGGTTTCGCAGAACGTTGAT
Pdgfra	GTCACAGTGCTGGAAGTGGT	ATGTGCCTGCCTTCGATCTC
MHC	CAAGACTGTCCGGAATGACA	GGCTTCTTGTTGGACAGGAT
Nkx2.5	CTGTCGCTTGCACTTGTAGC	GACAAAGCCGAGACGGATGG
Hand1	TGAACTCAAAAAGACGGATGG	CTTTAATCCTCTTCTCGCCG
Bmp2	TCTTCCGGGAACAGATACAGG	TGGTGTCCAATAGTCTGGTCA
Myogenin	CTACAGGCCTTGCTCAGCTC	ACGATGGACGTAAGGGAGTG
Mck	CACCATGCCGTTCGGCAACA	GGTTGTCCACCCCAGTCT
Omd	GGTGAGCAGAGGAGTACTAACGG	TGTTGTCCTGACTGTCATGGTCGT
Col6a2	GGGGGTGGTCAACTTCGCCG	CGGATGCCCTCTTCACGGGC
Id1	GCGAGATCAGTGCCTTGG	CTCCTGAAGGGCTGGAGTC
Bmp2 ChIP-1	GTCCGGAGCACAGTCTTACC	CGGGCCAAGTGTCTTTTGTG
Bmp2 ChIP-2	CTTGCCGGGAGAGTGACTTG	TCCGGGTGTTCTCCCTGTAG
Gsc	TTGCACAGACAGTCGATGCTACT	TCGTTGCTTTCTCGACCCC
Mixl1	CGGTTCTGGATCATCTCTCAA	TACCGAGAACAAGCCAGCAGT
Tubb3	GGCAACTATGTAGGGGACTCAG	CCTGGGCACATACTTGTGAG
Pax6	ACCCGGCAGAAGATCGTAG	TTTGCATCTGCATGGGTCT
Bmp2 R-loop	GTCCGGAGCACAGTCTTACC	CGGGCCAAGTGTCTTTTGTG

Supplementary Table S2. List of oligonucleotides.

Primer	Sequence (5’-3’)
RACE	
Universal adapter	AGCAGTGGTATCAACGCAGAGTAC
SMARTer II A Oligo	AAGCAGTGGTATCAACGCAGAGTACGCGGG
3'RACE-F	CAACCCAATGAGGGGGTCTC
5'RACE-inner-R	CCAACTCAGGTCCCCTGAAAG
5'RACE-outer-R	TGCCGTGGAGACCAGAAGAA
KIpA generation	
Up-F	GGGATCCTCTAGAGATTCATGCGCCAGTCTCCCAG
Up-R	CTCCATGCCCCCAGCTCTTCCGATTTCC
PolyA-F	GAGCTGGGGGCATGGAGCTCGAGACGCGTGGATC
PolyA-R	CATTCCGCTCCAAACTCGCCCGTCCGCCTCAGAAGCCATAGAG
Down-F	CGGGCGAGTTTGGAGCGG
Down-R	GCCTGCAGGTCGACGATCCCCTTCCCGTTCCTTTCTA
DNA templates	
lncRNA-Smad7 template-F	ATCCTAATACGACTCACTATAGGAG
lncRNA-Smad7 template-R	ACTTGATCCAGTGTTTATTTAGCAT
sgRNAs for *lncRNA-Smad7*	
KI-sgRNA-F	CACCGGGCATGGAGCGGGCGAGTT
KI-sgRNA-R	AAACAACTCGCCCGCTCCATGCCC
KO-5'sgRNA-F	CACCGGGCATGGAGCGGGCGAGTT
KO-5'sgRNA-R	AAACAACTCGCCCGCTCCATGCCC
KO-3'sgRNA-F	CACCGGACATGGCGGGTGTTTGGC
KO-3'sgRNA-R	AAACGCCAAACACCCGCCATGTCC
shRNAs	
sh*lncRNA-Smad7*-1-F	CCGGGCATTTCTCAAGCCATGTTTACTCGAGTAAACATGGCTTGAGAAATGCTTTTTG
sh*lncRNA-Smad7*-1-R	AATTCAAAAAGCATTTCTCAAGCCATGTTTACTCGAGTAAACATGGCTTGAGAAATGC
sh*lncRNA-Smad7*-2-F	CCGGAGCGCCGATTTCAGACTATTCCTCGAGGAATAGTCTGAAATCGGCGCTTTTTTG
sh*lncRNA-Smad7*-2-R	AATTCAAAAAAGCGCCGATTTCAGACTATTCCTCGAGGAATAGTCTGAAATCGGCGCT
sh*Bmp2*-F	CCGGGTATAATGGTCAGAGTTATTTCTCGAGAAATAACTCTGACCATTATACTTTTTG
sh*Bmp2*-R	AATTCAAAAAGTATAATGGTCAGAGTTATTTCTCGAGAAATAACTCTGACCATTATAC
sh*Hand1*-F	CCGGGAAGACACACCCTTCCAACCCCTCGAGGGGTTGGAAGGGTG

	TGTCTTCTTTTTG
sh*Hand1*-R	AATTCAAAAAGAAGACACACCCTTCCAACCCCTCGAGGGGTTGGAA GGGTGTGTCTTC

Supplementary Table S3. ChIRP probes of *lncRNA-Smad7* used for this study.

No.	Sequence (5'-3')
#1	ACTTGATCCAGTGTTTATTTAGCATATACTGAGTCTCATCAAATATTTTCAGAGTGCG
#2	AACCTGCCAAACACCCGCCATGTCCCCAGATCCTTGGTTGCTGGGGCCCTGGGTGCAG
#3	GAGAACAGAAGATGCATGTCCTCACCTGGTCACAGTACATGACTCTGGGGGCTGCAATA
#4	CATTCTGGAAACACAGCTCAAGGCAAGGTGTCCTCTTTTTGCAGAGCTCTGGAGC
#5	GAGCCAAGCTCTCTGAGACAGCTGTTCATTCTTCAGTGTGGCAGTGGACAGACGAATC
#6	AGGTGAGTTTGGGGCCAGAAAAAAAGCTATCCCAAGAGTTGGAGTCCGCCAAACTAG
#7	GTAAGGAAAAGGAAGCTGGAAGATTTTGAGTTAATTTCCCTCAGCTTTTGGAGTTATC
#8	GAGTTATCATTGTTCTCATCATCGACATATCTAGATTCTTCTACTGTCTTTGGTCATTC
#9	CTGAAATAGTGCCTCAACTTCTCAGTTTTGGATCAAGAGACCCCCTCATTGGGTTGC
#10	CCAGCATTTAACCTTGCTGAATAGCTCCTGTAAATAATCACCATCAAAGACACACCAAG
#11	GCGGCTTGGCTCAGTGGATAAAGGCACTGGCCAACAAGCCTGATGACTTGGGTTTGCCC
#12	CGTGGAACCCACATGGTAGATGGAGAGGGGTGGGACTGGGGAGGTGGCAGGCATTAATC
#16	GCAAGTTCAAGGCCAGGCTGGTTTACATAAGTTCCAGGACAGCCAGGGCTGCATAATAC
#17	CTGTCTTAAAAAGAGGCGGGGCAAGGAGAGGGAGCACAAGGGCAGCTGTGCAGAG
#18	CAGAGGCATAGTGAAAGAGGGGTGATTCAGTCTCAGAGACTGGGGACCTGTGCAGATG
#19	GCAGATGGAGAGAATACTGCAGGGTGAGTCAACACACAC
#20	CACTCACCCCCAACCCTGCTCCCCCAGTTCTGGAGCTGCAGCAGGAACCCTAAGGTGTC
#21	GTGTCTGGGAAAGGGGGCAAACTCCAGGCCACTAGGACTTCACAGGGTGGCCAAAGACC
#22	CCTTGGGTGGGGACACAGTGAGATCTTTGATAAGCAGTCATTGGGACAGAGGAGCTCTG
#23	GGCATCTGAGAAAGACCAGAAATGGGGAGAGGAAAGCAGACCGGTGCTGAGGTTGTG
#24	GTGTTCCGTACCCTGGCTGTGTTCCACGTAGGCCAGGGGAATAAACATGGCTTGAG
#25	GAAATGCTTCAAAGATGACAGACTCAGTGTTGTGTGAAGGAGGGTGAGGCAGAGGTTG

#26	GTCCTTTGTGTCTGGTTCTTACATCAGGAGAAGTAGCTTCTGGAATAAGGTTGGCTTGG
#27	GCCATCTGGGACAGCAGAGCTAAGGTGCCTTTGCAGTGTCATGTCGGCTGATGGTTAC
#28	GTTACGTGGGCCTGAAGTTCAGAGCGAAGGCCAAGTGTTCCTCCTGAGCCCTGTATC
#29	CTTCAGCTACAGTGGTAGCTCACAGAGAAGTGCGTGCTGGAATCTGGGGAGCTATGTC
#30	GTCTTTCTTCTCAGGGTGTCTGTGTTCTTCAAGCATGTACCACCAGACGTGCATCAAC
#31	CAACAATCAATAAGATAGAAAACGTAAGTTTAGTCACATCTTGTTAGAACCCCGAGCCC
#32	CCTATGTGAACTTGACCATGAATAGTGGAGTTTGGATTGGAGCCAGTTCGCAGGTGCTG
#33	GAGACTCTTCGAGACCCTTTGCAGCAAGCGCCACTCCCATTTTCTTCCATCCTCCTC
#34	GTTTCCAGACTTCTCCAGGTTGTCCACGTAGCCACGAAGCGTAATTAGAAGGCTGCCC
#35	CTTTGCTTTCAGTTTATTCAATTCTTCCTGTCACCTGCAGCCTGGTTTAGCCTCTCTTG
#36	CTGAGGTCCACTGTTTGGTCAAGCCCTGAGCCGCCGCAGAACTCCTCCATGGAATCAC
#37	CTTCTTAAAACAGGCTTCCTCGTCGGGTGTGGTGGTGCACACCTTTAATCTCAGCACTC
#38	CTCGGGAGTCAGAGGCAGGCGGATTTCTGAGTTCAAGGCCAGCCTGGTCTACAAGTTCC
#39	GTTCCAGGACAGCCAGGGCTATACAGAGAAACCCTGTCTCAAAAACAAAACAAAAC
#40	CAAACAAAAAAAACCCCCAAAACAAACAAACAAACAAAAAATCAGGCTTCCTATGC
#42	GAACGCAGTGCCGTGGAGACCAGAAGAATCCAGAATCTCCCGGAGCTGGAGTTACATGC
#43	CAGTTAGGAGGTACCTGAAGACATTAGGAGGCCAACTCAGGTCCCCTGAAAGAGCATCC
#44	CCACTGCTCTTTACTTCTGAGCCACCTCTCCAGCCCCTTCCCGTTCCTTTCTAAGATG
#45	GACACATTTCCTAGACTACATTTCTGTCTCTTTGGTCAGTCCTTCCCTGCCTATAATGG
#46	TGGTGGGGTCATCCCTCCTCCATTTCCTTCCTTGGTCTTCTTGGCTGCTGTCTTCTTCC
#47	CCGGGTTGCTTTAGAGTGTTCACACACCATTCCAATAAGTGGCATTTTAATTTTTAG
#48	GTAGAATTACTATGCCATCCAGTAATTACTGCTTACGTGCCCCTCTCTGTCCGGTCCTG
#49	CCTGCTGATGGTCCTATACTGTTGTCTTCCTTCCACAACGTAGGGCTCC

#50	GCTCATTCCGCTCCAAACTCGCCCGCTCCATGCCCCCAGCTCTTCCGATTTCCCCCTGC

Supplementary Table S4. Antibodies used for this study.

Antibodys	SOURCE	IDENTIFIER
Mouse monoclonal to GAPDH	ZSGB-BIO	Cat# TA-08; RRID: AB_2747414
Mouse monoclonal to cardiac Troponin T	Abcam	Cat# ab8295; RRID: AB_306445
Rabbit polyclonal to phospho-Histone H3 (Ser10)	Abclonal	Cat# AP0840; RRID: AB_2771171
Rabbit monoclonal to tri-Methyl-Histone H3 (Lys27)	Cell Signaling Technology	Cat# 9733; RRID: AB_2616029
Rabbit monoclonal to phospho-Smad2/Smad3	Cell Signaling Technology	Cat# 8828S; RRID: AB_2631089
Rabbit monoclonal to phospho-Smad1/Smad5/Smad9	Cell Signaling Technology	Cat# 13820; RRID: AB_2493181
Rabbit monoclonal to Smad2	Cell Signaling Technology	Cat# 5339; RRID: AB_10626777
Rabbit monoclonal to Smad1	Cell Signaling Technology	Cat# 9743; RRID: AB_2107780
Mouse Monoclonal to MY-32	Abcam	Cat# ab51263 RRID: AB_2297993
Mouse monoclonal to hnRNPK	Thermo Fisher	Cat# MA5-36291; RRID: AB_2896813

Supplementary Table S5. A summarized list of representative significant genes responsive to TGF-β signaling in both hESCs and mESCs is provided as a separate file (.xlsx format). The genes containing more than five CA- or GU- repeats in the exon regions were picked out, and analyzed with the RNA-seq data (2-fold cutoff) in both hESCs (DMSO/SB431542) (PMID: 32398665, GSE133630) and mESCs (Activin A/SB431542) (PMID: 31564646, GSE115169).

Supplementary Table S6. A summarized list of ChIRP-MS for the representative significant proteins interacted with lncRNA-Smad7 in mESCs is provided as a separate file (.xlsx format).

SUPPLEMENTARY REFERENCES

1. Arase, M., Horiguchi, K., Ehata, S., Morikawa, M., Tsutsumi, S., Aburatani, H., Miyazono, K. and Koinuma, D. (2014) Transforming growth factor-beta-induced lncRNA-Smad7 inhibits apoptosis of mouse breast cancer JygMC(A) cells. *Cancer Sci*, **105**, 974-982.
2. Song, C., Wang, J., Ma, Y., Yang, Z., Dong, D., Li, H., Yang, J., Huang, Y., Plath, M., Ma, Y. *et al.* (2018) Linc-smad7 promotes myoblast differentiation and muscle regeneration via sponging miR-125b. *Epigenetics*, **13**, 591-604.
3. Xi, Q., Wang, Z., Zaromytidou, A.I., Zhang, X.H., Chow-Tsang, L.F., Liu, J.X., Kim, H., Barlas, A., Manova-Todorova, K., Kaartinen, V. *et al.* (2011) A poised chromatin platform for TGF-beta access to master regulators. *Cell*, **147**, 1511-1524.
4. Murry, C.E. and Keller, G. (2008) Differentiation of embryonic stem cells to clinically relevant populations: lessons from embryonic development. *Cell*, **132**, 661-680.
5. Consortium, E.P. (2012) An integrated encyclopedia of DNA elements in the human genome. *Nature*, **489**, 57-74.
6. Schertzer, M.D., Braceros, K.C.A., Starmer, J., Cherney, R.E., Lee, D.M., Salazar, G., Justice, M., Bischoff, S.R., Cowley, D.O., Ariel, P. *et al.* (2019) lncRNA-Induced Spread of Polycomb Controlled by Genome Architecture, RNA Abundance, and CpG Island DNA. *Mol Cell*, **75**, 523-537 e510.
7. Ying, Q.L., Stavridis, M., Griffiths, D., Li, M. and Smith, A. (2003) Conversion of embryonic stem cells into neuroectodermal precursors in adherent monoculture. *Nat Biotechnol*, **21**, 183-186.
8. Guo, X., Xu, Y., Wang, Z., Wu, Y., Chen, J., Wang, G., Lu, C., Jia, W., Xi, J., Zhu, S. *et al.* (2018) A Linc1405/Eomes Complex Promotes Cardiac Mesoderm Specification and Cardiogenesis. *Cell Stem Cell*, **22**, 893-908 e896.
9. Yin, Y., Yan, P., Lu, J., Song, G., Zhu, Y., Li, Z., Zhao, Y., Shen, B., Huang, X., Zhu, H. *et al.* (2015) Opposing Roles for the lncRNA Haunt and Its Genomic Locus in Regulating HOXA Gene Activation during Embryonic Stem Cell Differentiation. *Cell Stem Cell*, **16**, 504-516.
10. Bolger, A.M., Lohse, M. and Usadel, B. (2014) Trimmomatic: a flexible trimmer for Illumina sequence data. *Bioinformatics*, **30**, 2114-2120.
11. Langmead, B. and Salzberg, S.L. (2012) Fast gapped-read alignment with Bowtie 2. *Nat Methods*, **9**, 357-359.
12. Ramirez, F., Ryan, D.P., Gruning, B., Bhardwaj, V., Kilpert, F., Richter, A.S., Heyne, S., Dundar, F. and Manke, T. (2016) deepTools2: a next generation web server for deep-sequencing data analysis. *Nucleic Acids Res*, **44**, W160-165.
13. Thorvaldsdottir, H., Robinson, J.T. and Mesirov, J.P. (2013) Integrative Genomics Viewer (IGV): high-performance genomics data visualization and exploration. *Brief Bioinform*, **14**, 178-192.
